# Discovery of secondary sulphonamides as IDO1 inhibitors with potent antitumour effects *in vivo*

**DOI:** 10.1080/14756366.2020.1765165

**Published:** 2020-05-28

**Authors:** Shushan Ge, Haiqing Zhong, Xuewei Ma, Yingbo Zheng, Yi Zou, Fang Wang, Yan Wang, Yue Hu, Yuezhen Li, Wen Liu, Wenjie Guo, Qiang Xu, Yisheng Lai

**Affiliations:** a State Key Laboratory of Natural Medicines, Jiangsu Key Laboratory of Drug Discovery for Metabolic Diseases, Center of Drug Discovery, China Pharmaceutical University, Nanjing, PR China; b State Key Laboratory of Pharmaceutical Biotechnology, School of Life Sciences, Nanjing University, Nanjing, PR China; c Department of Organic Chemistry, School of Science, China Pharmaceutical University, Nanjing, PR China

**Keywords:** Indoleamine 2,3-dioxygenase 1, kynurenine pathway, immune escape, immune checkpoint, secondary sulphonamides

## Abstract

Indoleamine 2,3-dioxygenase 1 (IDO1) as a key rate-limiting enzyme in the kynurenine pathway of tryptophan metabolism plays an important role in tumour immune escape. Herein, a variety of secondary sulphonamides were synthesised and evaluated in the HeLa cell-based IDO1/kynurenine assay, leading to the identification of new IDO1 inhibitors. Among them, compounds **5d**, **5l** and **8g** exhibited the strongest inhibitory effect with significantly improved activity over the hit compound BS-1. The *in vitro* results showed that these compounds could restore the T cell proliferation and inhibit the differentiation of naïve CD4^+^ T cell into highly immunosuppressive FoxP3^+^ regulatory T (Treg) cell without affecting the viability of HeLa cells and the expression of IDO1 protein. Importantly, the pharmacodynamic assay showed that compound **5d** possessed potent antitumour effect in both CT26 and B16F1 tumours bearing immunocompetent mice but not in immunodeficient mice. Functionally, subsequent experiments demonstrated that compound **5d** could effectively inhibit tumour cell proliferation, induce apoptosis, up-regulate the expression of IFN-*γ* and granzyme B, and suppress FoxP3^+^ Treg cell differentiation, thereby activate the immune system. Thus, compound **5d** could be a potential and efficacious agent for further evaluation.

## 1. Introduction

With the increasing recognition of the interaction between the immune system and tumour cells, immunotherapy has gained tremendous attention in the field of cancer research^1^^,^^2^. The recent clinical successes of immune checkpoint inhibitors and chimeric antigen receptor T cell (CAR-T) therapy have dramatically changed the landscape of the cancer treatment^3–6^. To date, the US Food and Drug Administration (FDA) has approved six immune checkpoint inhibitors blocking cytotoxic T lymphocyte-associated antigen 4 (CTLA-4), programmed cell death protein 1 (PD-1) or programmed cell death ligand 1 (PD-L1), and the second CAR-T cell therapy for the treatment of solid and hematological malignancies. However, their limited response rate and immune-related adverse effects call for a need to develop other immunotherapy options to solve those problems^5–7^.

Indoleamine 2,3-dioxygenase 1 (IDO1) is a haem-containing enzyme that catalyses the initial and rate-limiting step in the catabolism of the essential amino acid tryptophan along the kynurenine pathway^8^. IDO1 plays an important role in the regulation of local inflammation and immune tolerance^9^. In cancer, aberrant activation of IDO1 results in suppression of antitumour immunity. The depletion of tryptophan and the accumulation of kynurenine and its downstream metabolites in the tumour microenvironment have been demonstrated to induce effector T cell anergy and enhance regulatory T (Treg) cell function, which eventually lead to an immunosuppressive milieu that helps tumour cells evade detection and killing by the immune system^10^. Tryptophan depletion has been implicated in the activation of general control non-derepressible 2 (GCN2) and suppression of the mechanistic target of rapamycin kinase (mTOR) pathways^11^^,^^12^. Furthermore, kynurenine and other tryptophan metabolites can activate the aryl hydrocarbon receptor (AHR)^13^. Indeed, high expression of IDO1 in tumour cells has been shown to correlate with worse clinical prognosis in patients with a variety of cancers, including colorectal, ovarian, endometrial, and hepatocellular carcinomas^14–18^. Moreover, preclinical studies have demonstrated that IDO inhibitors can effectively restore antitumour immunity and have the potential to synergize with chemotherapy, radiotherapy, and other immunotherapies, including anti-CTLA-4, anti-PD-1 and anti-PD-L1 antibodies^19–22^. Hence, these findings have motivated interest in developing IDO1 inhibitors for cancer immunotherapy.

**Figure 1.**
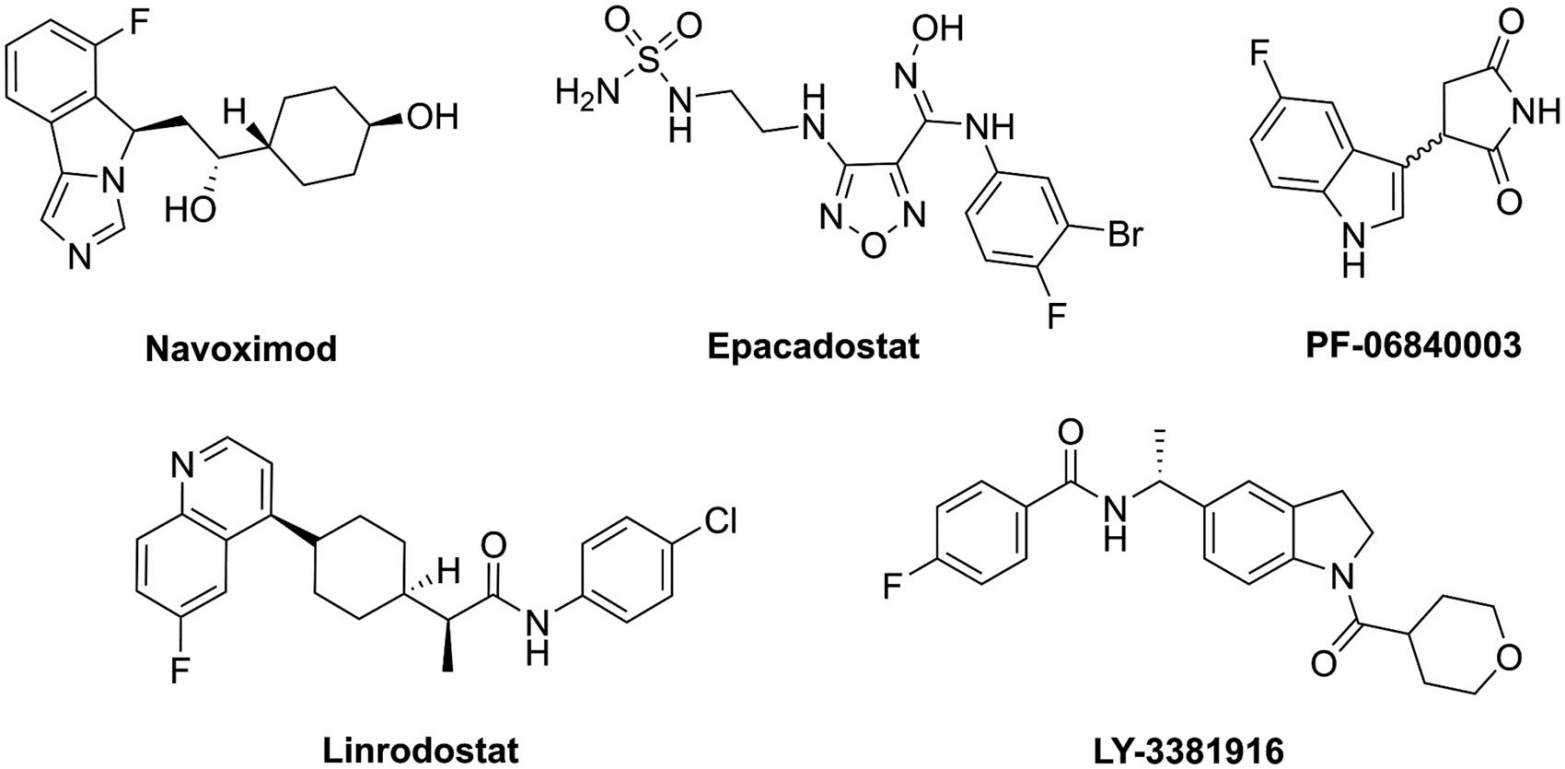
Structure of representative IDO1 clinical candidates.

So far, a number of IDO1 inhibitors have been identified from high-throughput screening^23^^,^^24^, structure-based design^25–27^, and natural product screening^28^^,^^29^. Several of them, including, navoximod, epacadostat, PF-06840003, linrodostat, LY-3381916 and KHK2455 (structure not disclosed), have entered clinical development as monotherapy and in combination with other immune therapies or chemotherapy (Figure 1)^30^. However, the FDA has not yet approved IDO inhibitors for the treatment of malignancies to date. We therefore need to continue to identify novel IDO1 inhibitors.

**Figure 2.**
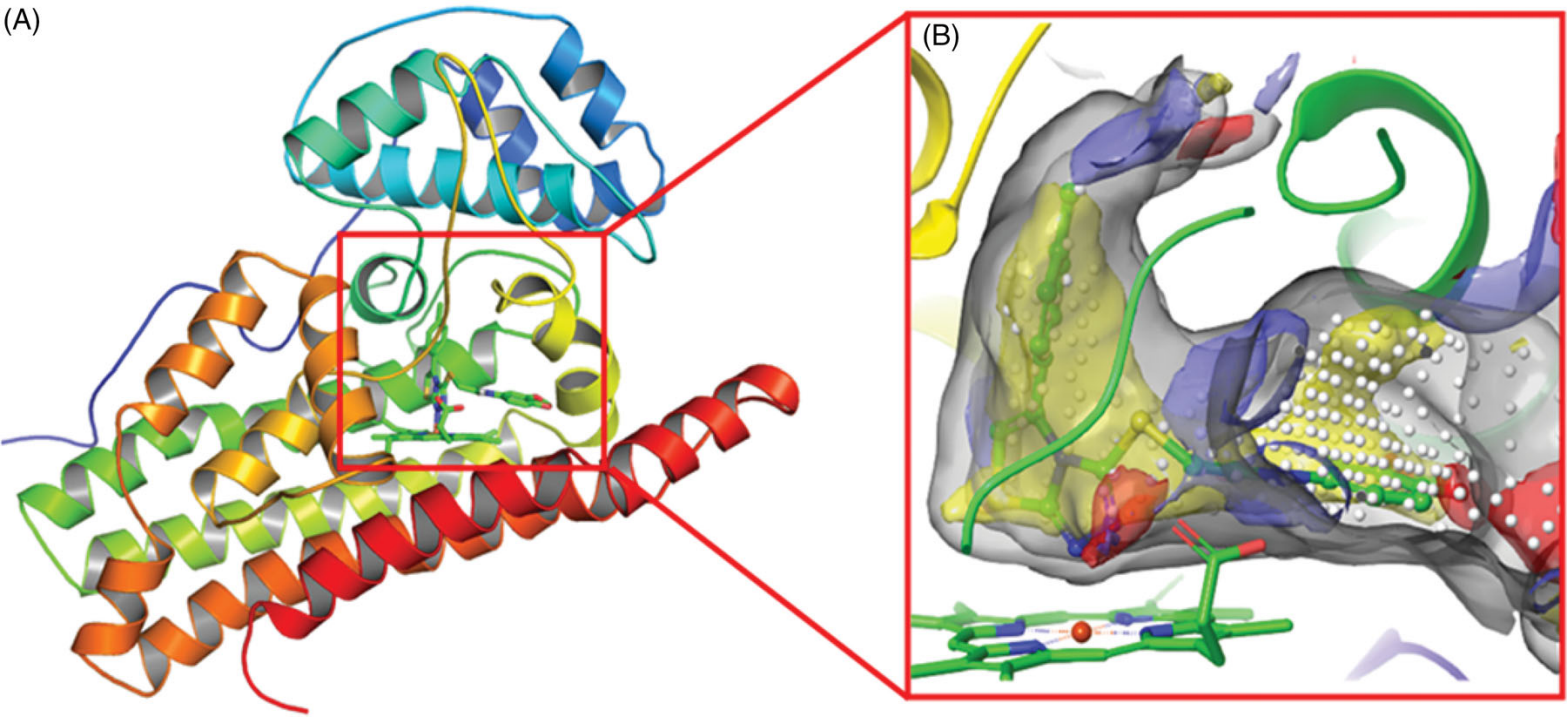
(A) The co-crystal of IDO1 (PDB ID: 4PK5). (B) Site maps generated using Sitemap: the ligand was Amg-1, white points represented the new identified active region, as well as yellow, blue and red solid maps represented hydrophobic regions, hydrogen bond donors and acceptors, respectively.

In recent years, we have been working on the development of novel IDO1 inhibitors^31–34^. From our previous study on the interactions between IDO1 and imidazole derivatives, it has been found that metal chelation, hydrogen bonding, and hydrophobic interactions between haem and the ligand contribute a large proportion to the binding affinity of the inhibitor^31^. Furthermore, the human IDO1 crystal structures in complex with the ligand Amg-1 (PDB ID: 4PK5) revealed that IDO1 is characterised by a small and highly lipophilic active site including two pockets (A and B), implying that the hydrophobic interaction may account for a large part of protein-ligand nonbonding interactions (Figure 2). In addition, Muller’s team demonstrated that as the charge on the coordinating atom decreased, the binding affinity to the haem iron increased, which would be possible to improve IDO1 inhibitory activity^35^. These findings provide directions for improving the binding affinity of novel IDO1 inhibitors.

In this study, we screened our in-house compound library using the HeLa cell-based IDO1/kynurenine assay, and found that the secondary sulphonamide compound BS-1 showed inhibitory activity against IDO1 with an IC_50_ value of 48.42 µM. As well known, bezenesulfonamide is a common pharmacophore that is the basis of several groups of drugs, such as sulpha antibiotics, thiazide diuretics and nonsteroidal antiinflammatory drug (NSAID) celecoxib^36^^,^^37^. Moreover, the sulphonyl is a heme-binding function that coordinates to the haem iron of IDO1^38^. Therefore, the hit compound BS-1 was further modified, as well as a variety of secondary sulphonamides were synthesised and evaluated in the HeLa cell-based IDO1/kynurenine assay. The resulting compounds **5d**, **5l** and **8g** showed high inhibitory activity without affecting the cell viability and the IDO1 protein expression. Subsequent *in vitro* and *in vivo* experiments demonstrated that compound **5d** could exert potent antitumour effects by activating the mouse immune system.

## 2. Material and methods

### 2.1. Chemistry

Melting points were determined on a RDCSY-I capillary apparatus and were uncorrected. Allmaterials used were commercially available and used as supplied. HG/T2354-92 silica gel 60 F254 sheets were used for analytical thin-layer chromatography (TLC). Column chromatography was performed on silica gel (300–400 mesh). ^1^H NMR spectra were recorded on a Bruker AV-300 spectrometer. Chemical shifts (δ) were given in parts per million (ppm) relative to the solvent peak. J values are in Hz. Chemical shifts are expressed in ppm downfield from internal standard TMS. Mass spectra (MS) were measured using a Thermo Scientific iCAP RQ ICP-MS. All the reagents and solvents were reagent grade and were used without further purification unless otherwise specified.

#### 2.1.1. General preparation of compounds 3a-i

To a solution of substituted aniline (0.97 mmol) in DCM (15 ml) was added triethylamine (1.22 mmol)^39^. A solution of 4-acrylamidobenzenesulfonyl chloride (0.81 mmol) in DCM (10 ml) was added dropwise to the mixture at 0 °C. The reaction was stirred at room temperature for 4 h. The solvent was evaporated under reduced pressure and the crude product was recrystallization to afford target compounds **3a-i**.

##### 2.1.1.1. N-(4-(N-Phenylsulfamoyl)phenyl)acetamide (3a)

White solid. Yield 90%. Mp 204–206 °C. ^1^H NMR (300 MHz, DMSO-*d_6_*) *δ* 10.17 (s, 1H), 10.04 (s, 1H), 7.54 (s, 4H), 7.08 (t, *J* = 7.8 Hz, 2H), 6.94–6.84 (m, 3H), 1.91 (s, 3H). MS (EI) *m/z* 289.1 [M-H]^−^.

##### 2.1.1.2. N-(4-(N-(p-Tolyl)sulfamoyl)phenyl)acetamide (3b)

White solid. Yield 87%. Mp > 250 °C. ^1^H NMR (300 MHz, DMSO-*d_6_*) *δ* 10.45 (s, 1H), 9.89 (s, 1H), 7.69 (d, *J* = 8.1 Hz, 2H), 7.57 (d, *J* = 8.4 Hz, 2H), 7.08 (d, *J* = 7.8 Hz, 2H), 6.73 (d, *J* = 7.8 Hz, 2H), 2.19 (s, 3H), 1.92 (s, 3H). MS (EI) *m*/*z* 303.1 [M-H]^−^.

##### 2.1.1.3. N-(4-(N-(4-Isopropylphenyl)sulfamoyl)phenyl)acetamide (3c)

Light yellow solid, Yield 90%, Mp 186–188 °C. ^1^H NMR (300 MHz, DMSO-*d_6_*) *δ* 10.16 (s, 1H), 9.91 (s, 1H), 7.54 (s, 4H), 6.95 (d, *J* = 8.1 Hz, 2H), 6.94 (d, *J* = 8.1 Hz, 2H), 2.65–2.58 (m, 1H),1.92 (d, *J* = 2.4 Hz, 3H), 0.97 (dd, *J* = 2.1, 2.7 Hz, 6H). MS (EI) m/z 331.1 [M-H]^−^.

##### 2.1.1.4. N-(4-(N-(3-Isopropylphenyl)sulfamoyl)phenyl)acetamide (3d)

White solid. Yield 90%. Mp 158–160 °C. ^1^H NMR (300 MHz, DMSO-*d_6_*) *δ* 10.17 (s, 1H), 9.95 (s, 1H), 7.55 (s, 4H), 6.98 (t, *J* = 7.7 Hz, 1H), 6.79–6.72 (m, 3H), 2.63–2.58 (m, 1H), 1.92 (s, 3H), 0.95 (d, *J* = 6.9 Hz, 6H). MS (EI) m/z 331.1 [M-H]^−^.

##### 2.1.1.5. N-(4-(N-Benzylsulfamoyl)phenyl)acetamide (3e)

White solid. Yield 90%. Mp 140–142 °C. ^1^H NMR (300 MHz, DMSO-*d*_6_) *δ* 10.20 (s, 1H), 7.89 (t, *J* = 6.3 Hz, 1H), 7.64–7.57 (m, 4H), 7.18–7.07 (m, 5H), 3.81 (d, *J* = 6.3 Hz, 2H), 1.96 (s, 3H). MS (EI) *m/z* 303.1 [M-H]^−^.

##### 2.1.1.6. N-(4-(N-(4-Chlorobenzyl)sulfamoyl)phenyl)acetamide (3f)

White solid. Yield 90%. Mp 172–174 °C. ^1^H NMR (300 MHz, DMSO-*d*_6_) *δ* 10.20 (s, 1H), 7.95 (t, *J* = 6.5 Hz, 1H), 7.63–7.56 (m, 4H), 7.17 (dd, *J* = 8.1, 8.4 Hz, 4H), 3.81 (d, *J* = 6.3 Hz, 2H), 1.95 (s, 3H). MS (EI) *m/z* 337.1 [M-H]^−^.

##### 2.1.1.7. N-(4-(N-(4-(Trifluoromethyl)benzyl)sulfamoyl)phenyl)acetamide (3g)

White solid. Yield 89%. Mp 186–188 °C. ^1^H NMR (300 MHz, DMSO-*d_6_*) *δ* 10.19 (s, 1H), 8.04 (t, *J* = 6.0 Hz, 1H), 7.62–7.56 (m, 4H), 7.50 (d, *J* = 8.1 Hz, 2H), 7.33 (d, *J* = 8.1 Hz, 2H), 3.93 (d, *J* = 5.7 Hz, 2H), 1.95 (s, 3H). MS (EI) m/z 371.1 [M-H]^−^.

##### 2.1.1.8. N-(4-(N-(3-(Trifluoromethyl)benzyl)sulfamoyl)phenyl)acetamide (3h)

White solid. Yield 88%. Mp 154–156 °C. ^1^H NMR (300 MHz, DMSO-*d_6_*) *δ* 10.19 (s, 1H), 8.05 (t, *J* = 6.3 Hz, 1H), 7.61–7.54 (m, 4H), 7.46–7.35 (m, 4H), 3.95 (d, *J* = 6.3 Hz, 2H), 1.95 (s, 3H). MS (EI) m/z 371.1 [M-H]^−^.

##### 2.1.1.9. N-(4-(N-Phenethylsulfamoyl)phenyl)acetamide (3i)

White solid. Yield 85%. Mp 130–132 °C. ^1^H NMR (300 MHz, DMSO-*d_6_*) *δ* 10.19 (s, 1H), 7.59 (q, *J* = 8.5 Hz, 4H), 7.46 (t, *J* = 5.9 Hz, 1H), 7.16–7.00 (m, 5H), 2.82–2.75 (m, 2H), 2.52 (t, *J* = 7.5 Hz, 2H), 1.95 (s, 3H). MS (EI) *m/z* 317.2 [M-H]^−^.

#### 2.1.2. General preparation of compounds 4a-f

To a solution of compounds **3** (0.68 mmol) in ethanol (15 ml) was added hydrochloric acid (1 ml)^39^. Then the mixture was stirred at 70 °C for 12 h. After the reaction was completed, the solvent was evaporated under reduced pressure. Water was added and the pH was adjusted to 7–8 with saturated NaHCO_3_ solution. The aqueous phase was extracted with EtOAc (3 × 30 ml). The combined organic layers were washed with water, brine, and dried. The solvent was removed *in vacuo* and the crude product was recrystallization to afford target compounds **4a-f**.

##### 2.1.2.1. 4-Amino-N-phenylbenzenesulfonamide (4a)

Light yellow solid. Yield 89%. Mp 188–190 °C. ^1^H NMR (300 MHz, DMSO-*d_6_*) *δ* 9.81 (s, 1H), 7.34 (d, *J* = 8.6 Hz, 2H), 7.15 (t, *J* = 7.8 Hz, 2H), 7.01 (d, *J* = 7.7 Hz, 2H), 6.94 (d, *J* = 7.3 Hz, 1H), 6.48 (d, *J* = 8.7 Hz, 2H), 5.92 (s, 2H). MS (EI) m/z 247.1 [M-H]^−^.

##### 2.1.2.2. 4-Amino-N-(p-tolyl)benzenesulfonamide (4b)

White solid. Yield 89%. Mp 188–190 °C. ^1^H NMR (300 MHz, DMSO-*d_6_*) *δ* 9.55 (s, 1H), 7.21 (d, *J* = 8.5 Hz, 2H), 6.83 (dd, *J* = 8.0, 8.3 Hz, 4H), 6.37 (d, *J* = 8.6 Hz, 2H), 5.82 (s, 2H), 2.03 (s, 3H). MS (EI) m/z 261.1 [M-H]^−^.

##### 2.1.2.3. 4-Amino-N-benzylbenzenesulfonamide (4c)

White solid. Yield 93%. Mp 116–118 °C. ^1^H NMR (300 MHz, DMSO-*d*_6_) *δ* 7.53 (t, *J* = 6.1 Hz, 1H), 7.32 (d, *J* = 8.6 Hz, 2H), 7.09–7.18 (m, 5H), 6.49 (d, *J* = 8.6 Hz, 2H), 3.73 (d, *J* = 5.9 Hz, 2H). MS (EI) *m/z* 261.1 [M-H]^−^.

##### 2.1.2.4. 4-Amino-N-(4-chlorobenzyl)benzenesulfonamide (4d)

White solid. Yield 95%. Mp 172–174 °C. ^1^H NMR (300 MHz, DMSO-*d*_6_) *δ* 7.66 (t, *J* = 6.2 Hz, 1H), 7.21–7.40 (m, 6H), 6.56 (d, *J* = 8.4 Hz, 2H), 5.91 (s, 2H), 3.83 (d, *J* = 6.2 Hz, 2H). MS (EI) *m/z* 295.1 [M-H]^−^.

##### 2.1.2.5. 4-Amino-N-(3-chlorobenzyl)benzenesulfonamide (4e)

White solid. Yield 90%. Mp 119–121 °C. ^1^H NMR (300 MHz, DMSO-*d*_6_) *δ* 7.70 (s, 1H), 7.42 (d, *J* = 8.6 Hz, 2H), 7.36–7.11 (m, 4H), 6.60 (d, *J* = 8.4 Hz, 2H), 5.92 (s, 2H), 3.90 (d, *J* = 5.0 Hz, 2H). MS (EI) *m/z* 295.1 [M-H]^−^.

##### 2.1.2.6. 4-Amino-N-phenethylbenzenesulfonamide (4f)

White solid. Yield 90%. Mp 138–140 °C. ^1^H NMR (300 MHz, DMSO-*d*_6_) *δ* 7.37 (d, *J* = 8.4 Hz, 2H), 7.14–7.25 (m, 5H), 6.56 (d, *J* = 8.5 Hz, 2H), 2.82 (m, 2H), 2.60 (t, *J* = 7.4 Hz, 2H). MS (EI) *m/z* 275.1 [M-H]^−^.

#### 2.1.3. General preparation of compounds 5a-m

To a solution of compounds **4** (0.55 mmol) in DCM (15 ml) was added TEA (1.1 mmol). Then acryloyl chloride (0.61 mmol) was added dropwise to the mixture at 0 °C for 0.5 h. The reaction was stirred at rt overnight. After the reaction was completed, the water was added to quench the reaction. The mixture was extracted with DCM to afford the crude product. The crude residue was recrystallization to afford target compounds **5a-m**.

##### 2.1.3.1. N-(4-(N-Benzylsulfamoyl)phenyl)acrylamide (5a)

White solid. Yield 75%. Mp 116–118 °C. ^1^H NMR (300 MHz, DMSO-*d*_6_) *δ* 10.50 (s, 1H), 8.02 (t, *J* = 6.2 Hz, 1H), 7.81 (d, *J* = 8.7 Hz, 2H), 7.72 (d, *J* = 8.7 Hz, 2H), 7.27–7.16 (m, 5H), 6.48–6.39 (m, 1H), 6.28 (d, *J* = 15.6 Hz, 1H), 5.79 (d, *J* = 9.9 Hz, 1H), 3.92 (d, *J* = 6.0 Hz, 2H). MS (EI) *m/z* 315.1 [M-H]^−^.

##### 2.1.3.2. N-(4-(N-(4-Methoxybenzyl)sulfamoyl)phenyl)acrylamide (5b)

White solid. Yield 81%. Mp 156–158 °C. ^1^H NMR (300 MHz, DMSO-*d*_6_) *δ* 10.38 (s, 1H), 7.82 (t, *J* = 6.0 Hz, 1H), 7.70 (d, *J* = 8.7 Hz, 2H), 7.61 (d, *J* = 8.7 Hz, 2H), 7.00 (d, *J* = 8.4 Hz, 4H), 6.69 (d, *J* = 8.7 Hz, 2H), 6.38–6.29 (m, 1H), 6.18 (d, *J* = 17.1 Hz, 1H), 5.69 (d, *J* = 9.9 Hz, 1H), 3.75 (d, *J* = 6.0 Hz, 2H), 3.57 (s, 3H). MS (EI) *m/z* 345.2 [M-H]^−^.

##### 2.1.3.3. N-(4-(N-(4-Chlorobenzyl)sulfamoyl)phenyl)acrylamide (5c)

White solid. Yield 80%. Mp 190–192 °C. ^1^H NMR (300 MHz, DMSO-*d*_6_) *δ* 10.40 (s, 1H), 7.98 (t, *J* = 6.3 Hz, 1H), 7.70 (d, *J* = 8.4 Hz, 2H), 7.61 (d, *J* = 8.4 Hz, 2H), 7.17 (dd, *J* = 8.4, 8.1 Hz, 4H), 6.37–6.28 (m, 1H), 6.18 (d, *J* = 16.5 Hz, 1H), 5.69 (d, *J* = 2.7 Hz, 1H), 3.82 (d, *J* = 6.0 Hz, 2H). MS (EI) *m/z* 349.1 [M-H]^−^.

##### 2.1.3.4. N-(4-(N-(3-Chlorobenzyl)sulfamoyl)phenyl)acrylamide (5d)

White solid. Yield 51%. Mp 157–159 °C. ^1^H NMR (300 MHz, DMSO-*d*_6_) *δ* 10.58 (s, 1H), 8.15 (s, 1H), 7.83 (dd, *J* = 9.0, 3.2 Hz, 2H), 7.78–7.65 (m, 2H), 7.47–7.06 (m, 4H), 6.44 (dd, *J* = 9.8, 3.4 Hz, 1H), 6.37–6.22 (m, 1H), 5.88 –5.74 (m, 1H), 3.99 (d, *J* = 3.3 Hz, 2H). 13C NMR (126 MHz, DMSO) *δ* 164.08, 142.99, 140.90, 135.28, 133.36, 131.94, 130.53, 128.38, 128.13, 127.79, 127.47, 126.65, 119.53, 45.86. MS (EI) *m/z* 349.1 [M-H]^−^.

##### 2.1.3.5. N-(4-(N-Phenethylsulfamoyl)phenyl)acrylamide (5e)

White solid. Yield 75%. Mp 154–156 °C. ^1^H NMR (300 MHz, DMSO-*d*_6_) *δ* 10.39 (s, 1H), 7.70 (d, *J* = 8.1 Hz, 2H), 7.61 (d, *J* = 7.5 Hz, 2H), 7.49 (s, 1H), 7.09 (m, 4H), 6.37–6.28 (m, 1H), 6.17 (d, *J* = 15.6 Hz, 1H), 5.69 (d, *J* = 8.7 Hz, 1H), 2.80 (s, 1H), 2.53 (s, 1H). MS (EI) *m/z* 353.1 [M + Na]^+^.

##### 2.1.3.6. N-(4-(N-(4-(Trifluoromethyl)benzyl)sulfamoyl)phenyl)acrylamide (5f)

White solid. Yield 80%. Mp 198–200 °C. ^1^H NMR (300 MHz, DMSO-*d*_6_) *δ* 10.38 (s, 1H), 8.07 (s, 1H), 7.69 (d, *J* = 7.8 Hz, 2H), 7.61 (d, *J* = 8.1 Hz, 2H), 7.50 (d, *J* = 7.2 Hz, 4H), 7.33 (d, *J* = 7.2 Hz, 2H), 6.37–6.15 (m, 1H), 6.18 (d, *J* = 16.5 Hz, 1H), 5.69 (d, *J* = 9.9 Hz, 1H), 3.94 (d, *J* = 4.5 Hz, 2H). MS (EI) *m/z* 407.1 [M + Na]^+^.

##### 2.1.3.7. N-(4-(N-(3-(Trifluoromethyl)benzyl)sulfamoyl)phenyl)acrylamide (5g)

White solid. Yield 77%. Mp 152–154 °C. ^1^H NMR (300 MHz, DMSO-*d*_6_) *δ* 10.55 (s, 1H), 8.19 (t, *J* = 6.2 Hz, 1H), 7.79 (d, *J* = 8.7 Hz, 2H), 7.69 (d, *J* = 8.7 Hz, 2H), 7.55–7.44 (m, 4H), 6.49–6.40 (m, 1H), 6.27 (d, *J* = 16.8 Hz, 1H), 5.78 (d, *J* = 3.9 Hz, 1H), 4.06 (d, *J* = 6.0 Hz, 2H). MS (EI) *m/z* 383.1 [M-H]^−^.

##### 2.1.3.8. N-(4-(N-(4-Fluorobenzyl)sulfamoyl)phenyl)acrylamide (5h)

White solid. Yield 82%. Mp 178–180 °C. ^1^H NMR (300 MHz, DMSO-*d*_6_) *δ* 10.40 (s, 1H), 7.95 (t, *J* = 6.0 Hz, 1H), 7.70 (d, *J* = 8.7 Hz, 2H), 7.61 (d, *J* = 8.4 Hz, 2H), 7.14 (t, *J* = 6.8 Hz, 1H), 6.97 (t, *J* = 8.6 Hz, 2H), 6.37–6.28 (m, 1H), 6.18 (d, *J* = 17.1 Hz, 1H), 5.70 (d, *J* = 9.9 Hz, 1H), 3.82 (d, *J* = 6.0 Hz, 2H). MS (EI) *m/z* 357.1 [M + Na]^+^.

##### 2.1.3.9. N-(4-(N-(3-Fluorobenzyl)sulfamoyl)phenyl)acrylamide (5i)

White solid. Yield 80%. Mp 152–154 °C. ^1^H NMR (300 MHz, DMSO-*d*_6_) *δ* 10.39 (s, 1H), 8.01 (t, *J* = 6.3 Hz, 1H), 7.70 (d, *J* = 8.4 Hz, 2H), 7.61 (d, *J* = 5.7 Hz, 2H), 7.22–7.15 (m, 1H), 6.95–6.89 (m, 3H), 6.37–6.28 (m, 1H), 6.18 (d, *J* = 16.8 Hz, 1H), 5.69 (d, *J* = 9.9 Hz, 1H), 3.87 (d, *J* = 6.3 Hz, 2H). MS (EI) *m/z* 357.1 [M + Na]^+^.

##### 2.1.3.10. N-(4-(N-(2-Chlorobenzyl)sulfamoyl)phenyl)acrylamide (5j)

White solid. Yield 78%. Mp 178–180 °C. ^1^H NMR (300 MHz, DMSO-*d*_6_) *δ* 10.41 (s, 1H), 8.00 (s, 1H), 7.71 (d, *J* = 7.5 Hz, 2H), 7.64 (d, *J* = 8.7 Hz, 2H), 7.28–7.15 (m, 4H), 6.39–6.29 (m, 1H), 6.18 (d, *J* = 17.4 Hz, 1H), 5.69 (d, *J* = 2.7 Hz, 1H), 3.91 (d, *J* = 4.5 Hz, 2H). MS (EI) *m/z* 349.1 [M-H]^−^.

##### 2.1.3.11. N-(4-(N-(4-Bromobenzyl)sulfamoyl)phenyl)acrylamide (5k)

White solid. Yield 83%. Mp 194–196 °C. ^1^H NMR (300 MHz, DMSO-*d*_6_) *δ* 10.44 (s, 1H), 7.98 (t, *J* = 6.2 Hz, 1H), 7.71 (d, *J* = 8.4 Hz, 2H), 7.61 (d, *J* = 8.7 Hz, 2H), 7.34 (d, *J* = 7.8 Hz, 2H), 7.07 (d, *J* = 7.8 Hz, 2H), 6.38–6.29 (m, 1H), 6.17 (d, *J* = 15.9 Hz, 1H), 5.69 (d, *J* = 10.2 Hz, 1H), 3.80 (d, *J* = 6.3 Hz, 2H). MS (EI) *m/z* 393.1, 395.1 [M-H]^−^.

##### 2.1.3.12. N-(4-(N-(3-Bromobenzyl)sulfamoyl)phenyl)acrylamide (5l)

White solid. Yield 81%. Mp 156–158 °C. ^1^H NMR (300 MHz, DMSO-*d*_6_) *δ* 10.40 (s, 1H), 8.01 (t, *J* = 6.0 Hz, 1H), 7.70 (d, *J* = 8.4 Hz, 2H), 7.60 (d, *J* = 8.7 Hz, 2H), 7.26 (s, 2H), 7.11 (d, *J* = 3.9 Hz, 2H), 6.38–6.29 (m, 1H), 6.18 (d, *J* = 15.9 Hz, 1H), 5.69 (d, *J* = 10.2 Hz, 1H), 3.86 (d, *J* = 6.0 Hz, 2H). MS (EI) *m/z* 393.1, 395.1 [M-H]^−^.

##### 2.1.3.13. N-(4-(N-(3,4-Dichlorobenzyl)sulfamoyl)phenyl)acrylamide (5m)

White solid. Yield 82%. Mp 148–150 °C. ^1^H NMR (300 MHz, DMSO-*d*_6_) *δ* 10.41 (s, 1H), 8.05 (s, 1H), 7.65 (d, *J* = 29.4 Hz, 4H), 7.34 (d, *J* = 30.0 Hz, 2H), 7.11 (s, 1H), 6.38–6.15 (m, 2H), 5.70 (s, 1H), 3.82 (d, *J* = 6.0 Hz, 2H). MS (EI) *m/z* 383.1 [M-H]^−^.

#### 2.1.4. General preparation of N-phenylacrylamide 6

To a solution of aniline (0.043 mol) in DCM (50 ml) was added TEA (0.086 mol). Then acryloyl chloride (0.047 mol) was added dropwise to the mixture at 0 °C for 0.5 h. The reaction was allowed to stir at rt overnight. After the reaction was completed, the water was added to quench the reaction. The mixture was extracted with DCM to afford the crude product that was purified by flash column chromatography on silica gel to afford N-phenylacrylamide **6** (white solid, Yield 72.8%)^40^. ^1^H NMR (300 MHz, Chloroform-d) *δ* 10.14 (s, 1H), 7.67 (d, *J* = 8.1 Hz, 2H), 7.32 (t, *J* = 7.8 Hz, 2H), 7.07 (t, *J* = 7.2 Hz, 1H), 6.44 (dd, *J* = 16.8, 9.9 Hz, 1H), 6.26 (dd, *J* = 16.8, 1.5 Hz, 1H), 5.75 (dd, *J* = 9.9, 1.5 Hz, 1H).

#### 2.1.5. General preparation of 4-acrylamidobenzenesulfonyl chloride 7

N-phenylacrylamide (0.031 mol) was added slowly to sulphuryl chloride (15 ml) at 0 °C for more than 0.5 h. The reaction was stirred at 70 °C for 1 h. After the reaction was completed, the mixture was poured into ice water slowly to quench the reaction. The mixture was stirred at rt for 1 h and the solid was filtered-off and washed with water (2 × 50 ml). The crude solid was dissolved in CH_2_Cl_2_ (50 ml), the organic layer was washed with brine, and the solvent was evaporated under reduced pressure to afford 4-acrylamidobenzenesulfonyl chloride as off-white solid (Yield 72.8%).

#### 2.1.6. General preparation of compounds 8a-j

To a solution of substituted aniline or heterocyclic amine (0.98 mmol) in DCM (15 ml) was added pyridine (1.22 mmol). A solution of 4-acrylamidobenzenesulfonyl chloride (0.81 mmol) in DCM (10 ml) was added dropwise to the mixture at 0 °C. The reaction was stirred at rt for 4 h. The solvent was evaporated under reduced pressure and the crude product was recrystallization to afford target compounds **8a-j**.

##### 2.1.6.1. N-(4-(N-Phenylsulfamoyl)phenyl)acrylamide (8a)

White solid. Yield 72%. Mp 212–213 °C. ^1^H NMR (300 MHz, Chloroform-d) *δ* 10.50 (s, 1H), 10.19 (s, 1H), 7.74 (dd, *J* = 23.7, 8.7 Hz, 4H), 7.22 (t, *J* = 7.8 Hz, 2H), 7.09–6.98 (m, 3H), 6.42 (dd, *J* = 16.8, 9.9 Hz, 1H), 6.28 (d, *J* = 15.3 Hz, 1H), 5.81 (d, *J* = 10.2 Hz, 1H). MS (EI) *m/z* 301.1 [M-H]^−^.

##### 2.1.6.2. N-(4-(N-(p-Tolyl)sulfamoyl)phenyl)acrylamide (8b)

White solid. Yield 79%. Mp 230–232 °C. ^1^H NMR (300 MHz, DMSO-*d*_6_) *δ* 10.46 (s, 1H), 9.98 (s, 1H), 7.75 (d, *J* = 8.7 Hz, 2H), 7.65 (d, *J* = 8.7 Hz, 2H), 6.96 (dd, *J* = 19.5, 8.4 Hz, 4H), 6.40 (dd, *J* = 17.1, 9.9 Hz, 1H), 6.26 (d, *J* = 17.1 Hz, 1H), 5.82–5.70 (m, 1H), 2.15 (s, 3H). MS (EI) *m/z* 315.1 [M-H]^−^.

##### 2.1.6.3. N-(4-(N-(4-Methoxyphenyl)sulfamoyl)phenyl)acrylamide (8c)

White solid. Yield 80%. Mp 234–235 °C. ^1^H NMR (300 MHz, DMSO-*d*_6_) *δ* 10.46 (s, 1H), 9.77 (s, 1H), 7.75 (d, *J* = 8.7 Hz, 2H), 7.60 (d, *J* = 8.7 Hz, 2H), 6.94 (d, *J* = 9.0 Hz, 2H), 6.77 (d, *J* = 8.7 Hz, 2H), 6.41 (dd, *J* = 17.1, 9.9 Hz, 1H), 6.26 (d, *J* = 15.3 Hz, 1H), 5.78 (d, *J* = 9.6 Hz, 1H), 3.64 (s, 3H). MS (EI) *m/z* 331.1 [M-H]^−^.

##### 2.1.6.4. N-(4-(N-(3-Methoxyphenyl)sulfamoyl)phenyl)acrylamide (8d)

White solid. Yield 77%. Mp 197–199 °C. ^1^H NMR (300 MHz, DMSO-*d*_6_) *δ* 10.38 (s, 1H), 10.09 (s, 1H), 7.63 (dd, *J* = 21.3, 8.7 Hz, 4H), 6.98 (t, *J* = 8.4 Hz, 1H), 6.53–6.51 (m, 2H), 6.45 (d, *J* = 8.4 Hz, 1H), 6.29 (dd, *J* = 16.8, 9.9 Hz, 1H), 6.15 (d, *J* = 16.8 Hz, 1H), 5.67 (d, *J* = 9.9 Hz, 1H), 3.52 (s, 3H). MS (EI) *m/z* 331.1 [M-H]^−^.

##### 2.1.6.5. N-(4-(N-(3-Fluorophenyl)sulfamoyl)phenyl)acrylamide (8e)

White solid. Yield 81%. Mp 212–215 °C. ^1^H NMR (300 MHz, DMSO-*d*_6_) *δ* 10.39–10.37 (m, 2H), 7.68 (d, *J* = 8.7 Hz, 2H), 7.60 (d, *J* = 8.7 Hz, 2H), 7.12 (t, *J* = 8.1 Hz, 1H), 6.97–6.91 (m, 3H), 6.29 (dd, *J* = 16.8, 9.6 Hz, 1H), 6.15 (d, *J* = 15.9 Hz, 1H), 5.68 (d, *J* = 9.3 Hz, 1H). MS (EI) *m/z* 319.1 [M-H]^−^.

##### 2.1.6.6. N-(4-(N-(3-Chlorophenyl)sulfamoyl)phenyl)acrylamide (8f)

White solid. Yield 83%. Mp 217–219 °C. ^1^H NMR (300 MHz, DMSO-*d*_6_) *δ* 10.39 (d, *J* = 8.1 Hz, 2H), 7.69 (d, *J* = 9.0 Hz, 2H), 7.61 (d, *J* = 9.0 Hz, 2H), 7.12 (t, *J* = 7.8 Hz, 1H), 6.97–6.92 (m, 3H), 6.30 (dd, *J* = 17.1, 10.2 Hz, 1H), 6.16 (d, *J* = 16.8 Hz, 1H), 5.70–5.62 (m, 1H). MS (EI) *m/z* 335.1 [M-H]^−^.

##### 2.1.6.7. N-(4-(N-(3-Chloro-4-fluorophenyl)sulfamoyl)phenyl)acrylamide (8g)

White solid. Yield 70%. Mp 224–226 °C. ^1^H NMR (300 MHz, DMSO-*d*_6_) *δ* 10.42 (s, 1H), 7.66 (d, *J* = 8.7 Hz, 2H), 7.56 (d, *J* = 8.4 Hz, 2H), 7.16 (t, *J* = 9.1 Hz, 1H), 7.05 (d, *J* = 4.5 Hz, 1H), 6.93–6.89 (m, 1H), 6.28 (dd, *J* = 16.5, 9.6 Hz, 1H), 6.15 (d, *J* = 16.8 Hz, 1H), 5.68 (d, *J* = 9.6 Hz, 2H). 13C NMR (126 MHz, DMSO) *δ* 164.19, 154.52 (d, *J*_C–F_ = 244.44 Hz, 1C), 143.66, 135.54, 133.30, 128.44, 128.38, 122.42, 121.28, 121.22, 120.13 (d, *J*_C–F_ = 6.3 Hz, 1 C), 119.60, 117.90 (d, *J*_C–F_ = 22.68 Hz, 1C). MS (EI) *m/z* 353.1 [M-H]^−^.

##### 2.1.6.8. N-(4-(N-(Pyrimidin-2-yl)sulfamoyl)phenyl)acrylamide (8h)

White solid. Yield 70%. Mp 229–230 °C. ^1^H NMR (300 MHz, DMSO-*d*_6_) *δ* 11.73 (s, 1H), 10.52 (s, 1H), 8.50 (d, *J* = 4.9 Hz, 2H), 7.95 (d, *J* = 9.0 Hz, 2H), 7.93 (d, *J* = 8.7 Hz, 2H), 7.05 (t, *J* = 4.9 Hz, 1H), 6.45 (dd, *J* = 17.0, 9.9 Hz, 1H), 6.30 (dd, *J* = 16.9, 2.1 Hz, 1H), 5.82 (dd, *J* = 9.8, 2.0 Hz, 1H). MS (EI) *m/z* 327.1 [M + Na]^+^.

##### 2.1.6.9. N-(4-(N-(3,4-Dimethylisoxazol-5-yl)sulfamoyl)phenyl)acrylamide (8i)

White solid. Yield 81%. Mp 206–208 °C. ^1^H NMR (300 MHz, DMSO-*d*_6_) *δ* 10.96 (s, 1H), 10.61 (s, 1H), 7.89–7.86 (d, *J* = 9.0 Hz, 2H), 7.74–7.71 (d, *J* = 9.0 Hz, 2H), 6.51–6.42 (m, 1H), 6.34–6.29 (d, *J* = 15.0 Hz, 1H), 5.85–5.82 (d, *J* = 9.0 Hz, 1H), 2.08 (s, 3H), 1.63 (s, 3H). MS (EI) *m/z* 320.1 [M-H]^−^.

##### 2.1.6.10. N-(4-(N-(5-Methylisoxazol-3-yl)sulfamoyl)phenyl)acrylamide (8j)

White solid. Yield 85%. Mp 232–233 °C. ^1^H-NMR (300 MHz, DMSO-*d*_6_) *δ* 11.35 (s, 1H), 10.56 (s, 1H), 7.87–7.80 (dd, *J* = 12.0, 9.0 Hz, 4H), 6.49–6.40 (m, 1H), 6.33–6.28 (d, *J* = 15.0 Hz, 1H), 6.13 (s, 1H), 5.85–5.81 (dd, *J* = 9.0, 3.0 Hz, 1H), 2.29 (s, 3H). MS (EI) *m/z* 306.1 [M-H]^−^.

### 2.2. Biological assays

The main bioassays performed in the study, including HeLa cell-based IDO1/kynurenine assay, Western blot analysis, T cell proliferation and Treg cell differentiation experiments, were carried out as described previously^32^.

#### 2.2.1. Cellular thermal shift assay

HeLa cells were seeded in 6-well culture plates at a density of 2 × 10^5^ per well. On the next day, human IFN-*γ* (100 ng/mL) were added and incubated for 24 h, and then the cells were treated with **5d** for 2 h. At the end of incubation, cells collected and subjected to CETSA assay. Briefly, incubated cells were equally divided into 10 parts, each part was heated for 3 min under different temperature (43, 46, 49, 52, 55, 58, 61, 64, 67, and 70 °C), and then the heated cells were kept at −80 °C for 12 h, then at room temperature for 5 min, and the process repeated one more time. After that, cell lysates were extracted by centrifugation at 20,000 × g, 20 min. Levels of IDO1 protein were assessed by Western blot analysis.

#### 2.2.2. *In vivo* antitumour activity assay

BALB/c mice (6–8 weeks old, 18–22 g) were supplied by Model Animal Research Centre of Nanjing University (Nanjing, China). Mice were maintained under standard specific-pathogen-free (SPF) conditions (21 ± 2 °C and 12-h light: dark cycle). Animal welfare and experimental procedures were carried out strictly in accordance with the Guide for the Care and Use of Laboratory Animals (The Ministry of Science and Technology of China, 2006) and the related ethical regulations of our university. All efforts were made to minimise animals’ suffering and to reduce the number of animals used. Mouse colon cancer cells (CT26) and melanoma cells (B16F1) were cultured and collected by centrifugation (1000 rpm, 5 min) and washed twice with ice-cold PBS. Then cells were diluted to 1 × 10^7^/ml and 1 × 10^6^ CT26/B16F1 cells (in 0.1 ml PBS) were injected subcutaneously into the right flanks of mice. All mice formed tumours three days after injection. Then mice were randomly distributed into five groups (*n* = 10/8) according to tumour volumes. **5d** (3.125, 6.5, 12.5 mg/kg) were administered (i.g) to each group respectively every day. 5-FU (25 mg/kg) and Cis-platinum (1 mg/kg) were administered (i.p) every three days. Tumour length and tumour width were measured with a Vernier calliper every three days. Tumour volumes were measured and calculated using the equation volume = a × b^2^/2, where “a” is the maximal width and “b” is maximal orthogonal width. After the administration were completed, mice were weighed, euthanized, and tumours were removed and the weight were taken.

Eight-week-old male BALB/c nude mice, weighing 18–22 g, were supplied by Model Animal Research Centre of Nanjing University (Nanjing, China). 1 × 10^7^/ml and 1 × 10^6^ CT26 cells (in 0.1 ml PBS) were injected subcutaneously into the right flanks of mice. After CT26 xenograft BALB/c nude mouse model was established, all mice were randomly divided into three groups (*n* = 8). **5d** (12.5 mg/kg) were administered (i.g) every day and 5-FU (25 mg/kg) awere administered (i.p) every three days. The following experimental procedures were similar to the described above.

#### 2.2.3. Immunohistochemistry analysis

For immunohistochemistry staining, the sections were deparaffinized, rehydrated, and washed in 1% PBS-Tween 20, and then treated with 2% hydrogen peroxide, blocked with 3% goat serum (Life Technology, 16210–064) and incubated for 2 h at room temperature with specific primary antibodies. Then the slides were incubated with streptavidin-HRP (Shanghai Gene Company, GK500705) for 40 min, then stained with DAB (Shanghai Gene Company, GK500705) substrate and counter-stained with haematoxylin. Images were acquired by microscopy (Olympus IX51).

### 2.3. Molecular modelling

In order to consider the flexibility of both ligand and protein, the induced fit docking (IFD) protocol in Schrödinger was employed. In IFD calculations, the ligands were first docked into the rigid receptor using softened energy function in *Glide*. By default, a maximum 20 poses per ligand were retained. Then, the protein degrees of freedom for each complex were sampled and the protein-ligand complexes were minimised. The protein structure in each pose now reflected an induced fit to the ligand structure and conformation. The best protein-ligand complex was then identified based on the predicted binding affinities of the docked ligand. Here, the residues within 5 Å of each of the 20 ligand poses were subjected to a conformational search and energy minimizations, and the residues outside this range were fixed. Finally, the minimised ligand was rigorously redocked into the induced-fit protein structure using Glide XP scoring mode, and metal constraints can be applied to both Glide docking stages in IFD protocol. The choice of the best-docked structure for each ligand was made using a model energy score that combines the energy grid score, the binding affinity predicted by GlideScore, and the internal strain energy for the model potential used to direct the conformational-search algorithm.

## 3. Results and discussion

### 3.1. Chemistry

**Scheme 1.**
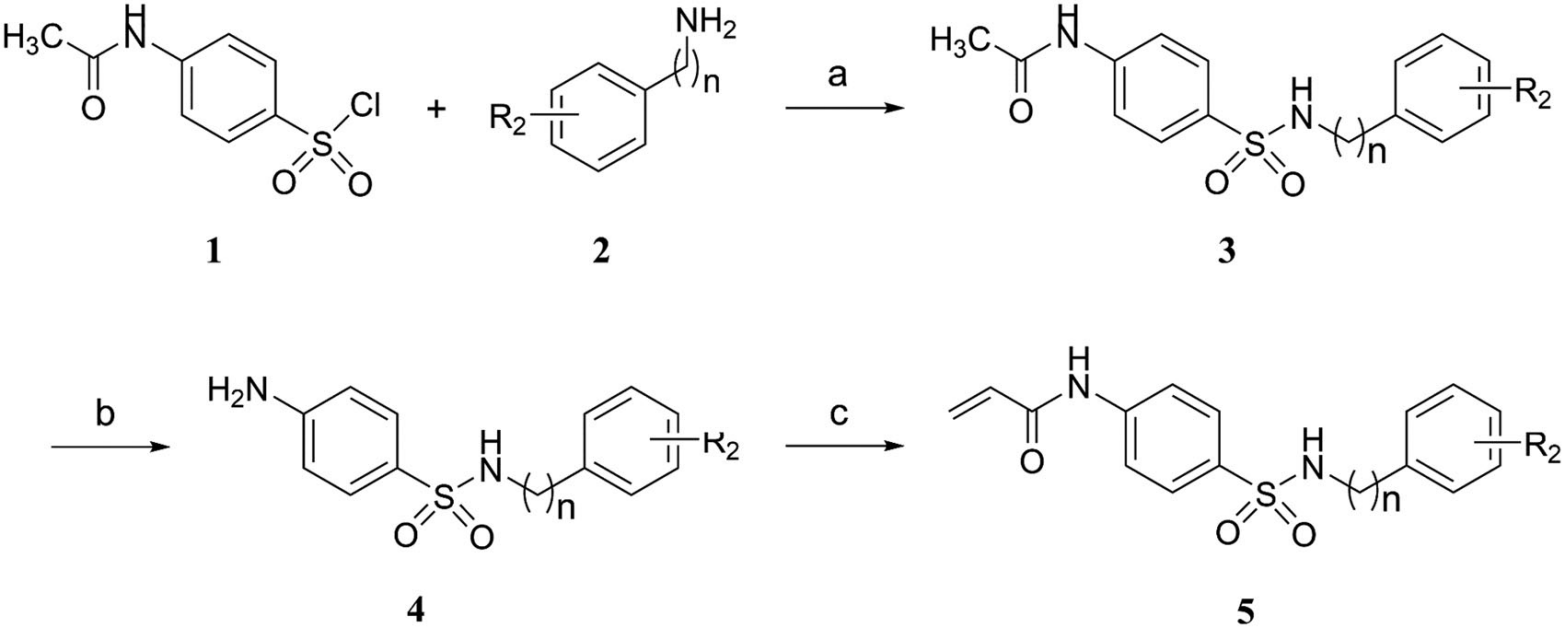
Reagents and conditions: (a) TEA, DCM, 0 °C, rt; (b) HCl, ethanol, 70 °C; (c) Acryloyl chloride, TEA, DCM, 0 °C, rt.

**Scheme 2.**
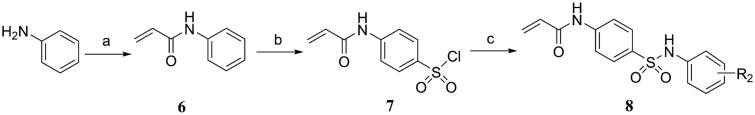
Reagents and conditions: (a) Acryloyl chloride, TEA, DCM, 0 °C, rt; (b) HSO_3_Cl, 70 °C, 1 h; (c) Substituted aniline, pyridine, DCM, 0 °C, rt.

The synthetic pathways for the target compounds were shown in Scheme 1 and 2. *N*-acetyl secondary sulphonamides **3** were prepared by the condensation reaction of substituted 4-acetamidobenzenesulfonyl chloride **1** with various amines **2**. Deacetylation of compounds **3** with concentrated HCl in ethanol gave amino secondary sulphonamides hydrochloride salts, and followed by neutralisation with saturated NaHCO_3_ solution to afford the corresponding amino secondary sulphonamides **4**. Finally, the compounds **5** were prepared via the condensation reaction of compounds **4** with acryloyl chloride (Scheme 1).

Alternatively, *N*-phenylacrylamide **6** was synthesised by condensation of aniline with acryloyl chloride in the presence of triethylamine, and followed by chlorosulfonation with chlorosulfonic acid to give 4-acetamidobenzenesulfonyl chloride **7**. Subsequently, the 4-acetamidobenzenesulfonyl chloride was treated with substituted aniline in the presence of pyridine to form the target compounds **8** (Scheme 2).

### 3.2. Effect of secondary sulphonamides on IDO1 activity

It is well known that IDO1 is broadly expressed in human cancers and is induced by pro-inflammatory cytokines, such as interferon gamma (IFN-*γ*) and tumour necrosis factor alpha (TNF-α)^41^. Here we utilised a human cervical cancer cell line HeLa that endogenously expresses IDO1 upon IFN-*γ* treatment to evaluate the IDO1 inhibitory activity of the synthesised compounds by measuring kynurenine secreted into the media. This cell-based assay, as a rapid and simple method for determination IDO1/kynurenine, is closely related to the potency of the enzyme assay and has been widely used to identify various IDO1 inhibitors^19^^,^^23^^,^^29^^,^^42^. In addition, cytotoxic compounds can reduce cell viability, which will decrease the IDO1 levels and then lower the conversion of tryptophan to kynurenine. Thus, we also employed this cellular assay to detect false positive IDO1 inhibitors and cytotoxic compounds by measuring HeLa cell viability. Clinical candidate navoximod was used as an assay control. The results were shown in Table 1 and 2.

**Table 1.** IDO1 inhibitory activity of the target compounds **3a-i**, **4a-f**, **5a-e** and **8a-b**.


Compd.	R_1_	*n*	R_2_	Inhibition %*	IC_50_ (μM)
BS-1	CH_3_CO	1	*m*-Cl	30.7%	49.42
**3a**	CH_3_CO	0	H	3.9%	N.D.**
**3b**	CH_3_CO	0	*p*-CH_3_	16.3%	N.D.
**3c**	CH_3_CO	0	*p*-CH(CH_3_)_2_	5.9%	N.D.
**3d**	CH_3_CO	0	*m*-CH(CH_3_)_2_	14.6%	N.D.
**3e**	CH_3_CO	1	H	2.5%	N.D.
**3f**	CH_3_CO	1	*p*-Cl	13.5%	N.D.
**3g**	CH_3_CO	1	*p*-CF_3_	14.5%	N.D.
**3h**	CH_3_CO	1	*m*-CF_3_	7.8%	N.D.
**3i**	CH_3_CO	2	H	5.9%	N.D.
**4a**	NH	0	H	12.0%	N.D.
**4b**	NH	0	*p*-CH_3_	10.8%	N.D.
**4c**	NH	1	H	7.4%	N.D.
**4d**	NH	1	*p*-Cl	–4.2%	N.D.
**4e**	NH	1	*m*-Cl	2.3%	N.D.
**4f**	NH	2	H	3.1%	N.D.
**5a**	CH_2_=CHCO	1	H	62.5%	22.36
**5b**	CH_2_=CHCO	1	*p*-OCH_3_	36.0%	40.06
**5c**	CH_2_=CHCO	1	*p*-Cl	40.4%	36.20
**5d**	CH_2_=CHCO	1	*m*-Cl	91.1%	6.26
**5e**	CH_2_=CHCO	2	H	55.2%	26.27
**8a**	CH_2_=CHCO	0	H	85.9%	13.18
**8b**	CH_2_=CHCO	0	*p*-CH_3_	25.3%	N.D.
Navoximod				98.6%	0.41

*Percentage inhibition at 30 μM. **N.D.: not determined.

**Table 2.** IDO1 inhibitory activity of the target compounds **5f-m** and **8c-j**.


Compd.	R_1_	*n*	R_2_	R_3_	Inhibition %*	IC_50_ (μM)
**5f**	CH_2_=CHCO	1	*p*-CF_3_	–	38.2%	39.16
**5g**	CH_2_=CHCO	1	*m*-CF_3_	–	49.5%	30.53
**5h**	CH_2_=CHCO	1	*p*-F	–	70.9%	20.54
**5i**	CH_2_=CHCO	1	*m*-F	–	37.8%	39.53
**5j**	CH_2_=CHCO	1	*o*-Cl	–	65.1%	22.66
**5k**	CH_2_=CHCO	1	*p*-Br	–	37.5%	42.08
**5l**	CH_2_=CHCO	1	*m*-Br	–	89.1%	6.64
**5m**	CH_2_=CHCO	1	*m,p*-diCl	–	44.0%	37.27
**8c**	CH_2_=CHCO	0	*p*-OCH_3_	–	20.9%	N.D.**
**8d**	CH_2_=CHCO	0	*m*-OCH_3_	–	74.8%	13.45
**8e**	CH_2_=CHCO	0	*m*-F	–	69.7%	17.66
**8f**	CH_2_=CHCO	0	*m*-Cl	–	89.5%	9.48
**8g**	CH_2_=CHCO	0	*m*-Cl-*p*-F	–	93.2%	3.76
**8h**	CH_2_=CHCO	0	–		16.4%	N.D.
**8i**	CH_2_=CHCO	0	**–**		23.6%	N.D.
**8j**	CH_2_=CHCO	0	–		22.7%	N.D.
Navoximod					98.6%	0.41

*Percentage inhibition at 30 μM. **N.D.: not determined.

Our group had already synthesised a series of *N*-acetyl secondary sulphonamides as the analogs of compound BS-1 and evaluated their inhibitory activity^43^. To our surprise, although most of these compounds had weak inhibitory activity against IDO1, N-(4-(N-(4-methoxybenzyl)sulfamoyl)phenyl)acetamide (HeLa IC_50_: 5.88 µM) showed good inhibitory activity, and its potency was remarkably increased by 8.4-fold as compared with the hit BS-1. Inspired by N-(4-(N-(4-methoxybenzyl)sulfamoyl)phenyl)acetamide, we further synthesised the analogues **3a-i** bearing an electron-donor group on the benzyl group, but they displayed only weak activity. Subsequently, we tried to remove the acetyl group of *N*-acetyl secondary sulphonamides, and found that all the obtained aniline compounds **4a-f** had no improvement in activity. These results indicated that a free amino group on the phenyl ring with strong electron-donating ability appeared to be unfavourable for the inhibitory activity of these compounds. To this end, we tried to introduce an acryloyl group instead of the acetyl group to the amine group. As expected, compounds **5a-e** bearing an acetyl group showed moderate to good inhibitory activity. Especially, compound **5d** (HeLa IC_50_: 6.26 µM) exhibited significantly stronger activity than the hit compound BS-1 (HeLa IC_50_: 49.42 µM) and its corresponding acetyl compound **4e** (HeLa IC_50_ > 50 µM).

We then further optimised the inhibitory activity of *N*-acryloyl secondary sulphonamides by modifying the substituents, including n, R_2_ and R_3_, while retaining the acryloyl group at R_1_. As shown in Table 2, most of *N*-acryloyl secondary sulphonamides displayed good inhibitory activity against IDO1. Among them, benzylamine derivative **5l** and aniline derivative **8g** exhibited potent activity with IC_50_ values of 6.64 µM and 3.76 µM, which were significantly increased by 7.4-fold and 13.1-fold as compared with the hit BS-1, respectively. It is worth noting that the compounds **5d**, **5g**, **5l**, and **8d** with a substituent at 3-position on aniline or benzylamine had higher activity than those compounds **5c**, **5f**, **5k** and **8c** with a substituent at 4-position (**5c** *vs* **5d**, **5f** *vs* **5g**, **5k** *vs* **5l, 8c** *vs* **8d**). However, compound **5i** (IC_50_: 39.53 µM) with a fluoro substituent at 3-position on aniline showed lower inhibitory activity compared with the corresponding compound **5h** with a fluorine atom at 4-position on aniline (IC_50_: 20.54 µM), which showed similar inhibitory activity with compound **5a** without substituent on aniline (IC_50_: 22.36 µM), likely due to the similar size of the fluorine and hydrogen atoms, Interestingly, compound **8g** containing a *m*-chlorine and a *p*-fluorine atoms showed the strongest potency (IC_50_: 3.76 µM), while compound **5m** with *m,p*-di-chlorine atoms had only moderate activity (IC_50_: 37.27 µM). It might be duo to the large volume of the 4-substituent on aniline or benzylamine that cause the bad clashes with the neighbouring residues in active site of IDO1. In addition, we also tried to replace benzene or benzyl rings with aromatic heterocycles (**8 h-8j**), including pyrimidine and isoxazole, but they showed only weak activity.

Taken together, compounds **5d**, **5l** and **8g** showed good inhibitory activity in the HeLa cell-based IDO1/kynurenine assay. Furthermore, the viability of HeLa cells was evaluated by the MTT method at the end of the assay. The readouts showed that most of the synthesised compounds did not affect cell viability under the experimental conditions (data not shown), indicating that IDO1 inhibitory activity of these compounds is not mediated by their cytotoxicity.

### 3.3. Effect of 5d, 5 l, 8f and 8g on IDO1 protein expression

**Figure 3.**
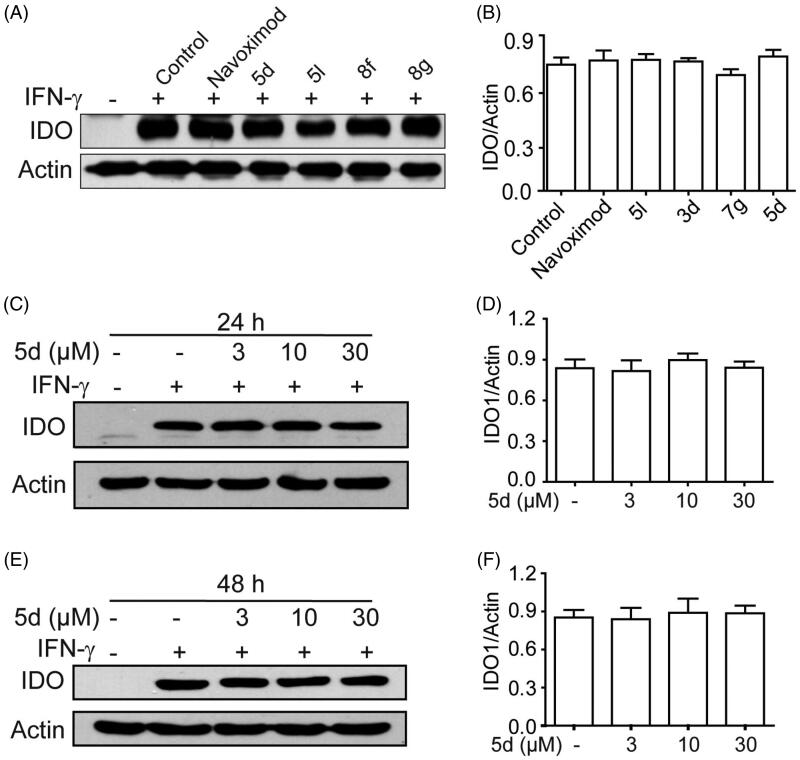
Compounds **3d**, **5d**, **5l** and **8g** did not affect the expression of IDO1 protein. (A) HeLa cells were treated with IFN-*γ* (100 ng/mL) for 24 h, then incubated with or without test compounds at their IC_50_ concentrations for 24 h, and expression of IDO was analysed by Western blot using an anti-IDO antibody. (B) The greyscale scanning statistical results of A. (C) HeLa cells were incubated with compound **5d** (3, 10, 30 μM) for 24 h. (D) The greyscale scanning statistical results of C. (E) HeLa cells were incubated with compound **5d** (3, 10, 30 μM) for 48 h. (F) The greyscale scanning statistical results of E. Data are presented as means ± SEM (*n* = 3).

To exclude the possibility that these secondary sulphonamides inhibit IDO1 activity by down-regulating IDO1 expression, we utilised Western blot to measure the expression of IDO1 protein in HeLa cells treated with different compounds. As depicted in Figure 3, compared with the blank group, the expression of IDO1 protein was significantly induced in IFN-*γ*–treated HeLa cells. Evidently, all tested compounds **5d**, **5l**, **8f** and **8g** did not influence IDO1 expression as compared with the control group. In addition, we further confirmed that treatment of HeLa cells with different concentrations of compound **5d** (3, 10, 30 µM) for 24 h or 48 h also did not affect IDO1 expression. The results demonstrated that these compounds blocked the kynurenine pathway by affecting IDO1 enzyme activity rather than its expression and/or HeLa cell viability.

### 3.4. Compounds 5d and 8g could effectively restore T cell proliferation

**Figure 4.**
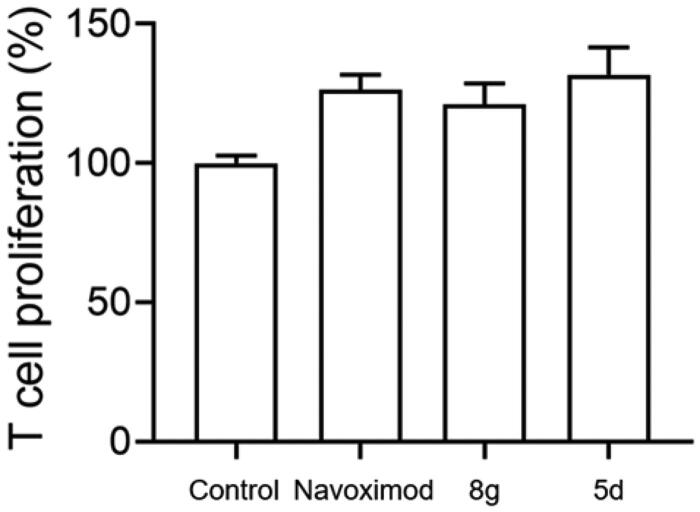
Compounds **5d** and **8 g** could promote the proliferation of T cells. Based on the B16F1-T cell co-culture system, use MTT assay to test T cells proliferation. The Navoximod and compounds were added to the system at the concentration of 1.5 and 18 μM. Data represent mean ± SEM (*n* = 3).

T lymphocytes are exceedingly sensitive to low tryptophan levels. Overactivation of the kynurenine pathway by high IDO1 expression leads to local tryptophan depletion, which can suppress T cell proliferation by activating the GCN2 kinase pathway and eventually result in T cell anergy^44^. Here, to determine whether secondary sulphonamides could rescue IDO1-mediated blockade of T cell proliferation, we used a co-culture of IDO1-expressing B16F1 melanoma cells with naïve CD4^+^ T cells to mimic the tumour microenvironment. Given that melanoma is a highly immunogenic tumour and IDO1 constitutively expresses in melanoma cells^40^, B16F1 murine melanoma cell lines that express IDO1 were then used to co-culture with T cells. Naïve CD4^+^ T cells were isolated from spleens of C57BL/6 mice. As shown in Figure 4, compounds **5d**, **8g** and navoximod displayed a significant augmentation on T cell activity stimulated with B16F1 cells. Especially, compound **5d** showed better enhancement of T cell proliferation than navoximod. These findings demonstrated that compounds **5d** and **8g** could reverse the suppression of T lymphocytes caused by IDO1.

### 3.5. Compounds 5d and 8g could effectively inhibit FoxP3^+^ Treg cell differentiation

**Figure 5.**
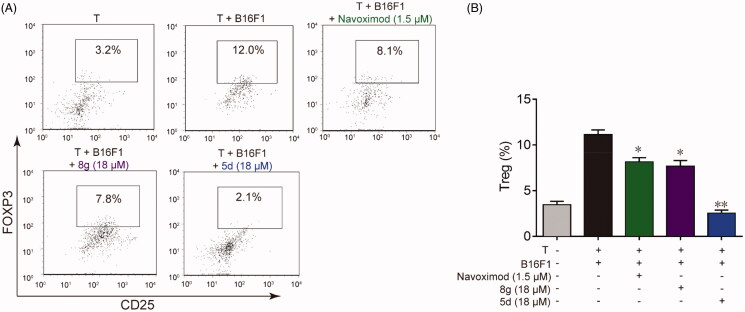
Compounds **5d** and **8 g** inhibited Treg cell differentiation in the cell co-culture system. (A) After co-cultured with B16F1 cells, T cells were collected. Surface staining was performed with CD4^+^ FTTC and CD25^+^ PE for 30 min at 4 °C. The cells were fixed and permeabilized with fixation buffer and permeabilization wash buffer. The intracellular staining was performed with FoxP3^+^ APC for 20 min. The cells were then analysed by flow cytometry analysis. (B) Statistical analysis of result A. Data are represented as ± SEM (*n* = 3). *p < 0.05, **p < 0.01 *vs* T + B16F1.

Besides causing local tryptophan depletion, high IDO1 expression can also accumulate metabolites of kynurenine pathway. The elevated metabolites can not only induce effector T cell death directly through their cytotoxicity, but also promote the differentiation of naïve CD4^+^ T cells into CD4^+^ FoxP3^+^ Treg cells by activating the AHR pathway^10^. As a highly immunosuppressive subset of CD4^+^ T cells, FoxP3^+^ Treg cells are critical to the maintenance of immune homeostasis and are involved in tumour immune escape, thereby contributing to tumour development and progression^45^. Therefore, we also utilised co-culture of B16F1 cells with naïve CD4^+^ T cells to test whether the synthesised compounds could suppress IDO1 mediated Treg cell differentiation. As illustrated in Figure 5, when the naïve CD4^+^ T cells were co-cultured with B16F1 cells under Con A stimulation for 48 h, the number of the FoxP3^+^ Treg cells increased approximately 4 times compared with naïve T cells cultured alone, indicating that IDO1-expressing B16F1 cells could significantly stimulate naïve T cell differentiation into FoxP3^+^ Treg cells. However, treatment with **5d**, **8g** and navoximod could significantly reduce FoxP3^+^ Treg cell differentiation. Especially, compound **5d** displayed the best effect in this test, and its FoxP3^+^ Treg cell number returned to the initial level. These data evidenced that compounds **5d** and **8g** could effectively suppress the differentiation of naïve CD4^+^ T cell into FoxP3^+^ Treg cells, which would help reverse IDO1-mediated immune suppression.

### 3.6. Compound 5d could bind to IDO1 protein in HeLa cells

**Figure 6.**
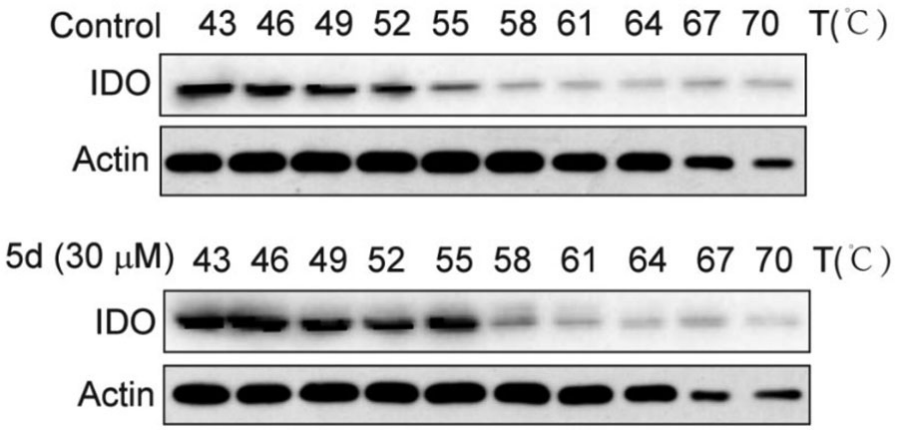
Compound **5d** could bind to IDO1 protein in HeLa cells. HeLa cells were treated with human IFN-*γ* (100 ng/mL) for 24 h, and then treated with **5d** for 2 h. The cells were collected and subjected to CETSA assay. The levels of IDO1 protein were assessed by Western blot analysis.

The cellular thermal shift assay (CETSA) is usually used to identify compounds and their potential targets. CESTA can detect the target engagement of drugs in a cellular context based on the biophysical principle of ligand-induced thermal stabilisation of target proteins^46^. To this end, we further employed the CETSA assay to detect whether compound **5d** could directly bind to the target protein IDO1 in HeLa cells. As presented in Figure 6, treatment with **5d** could improve the thermal stability of IDO1 protein in HeLa cells as compared with the vehicle control, and still maintained the protein stability at 55 °C. These results suggested that compound **5d** could enter HeLa cells and then directly bind to IDO1 protein.

### 3.7. Compound 5d could effectively inhibit colon carcinoma growth in immunocompetent mice, but not in immunodeficient mice

**Figure 7.**
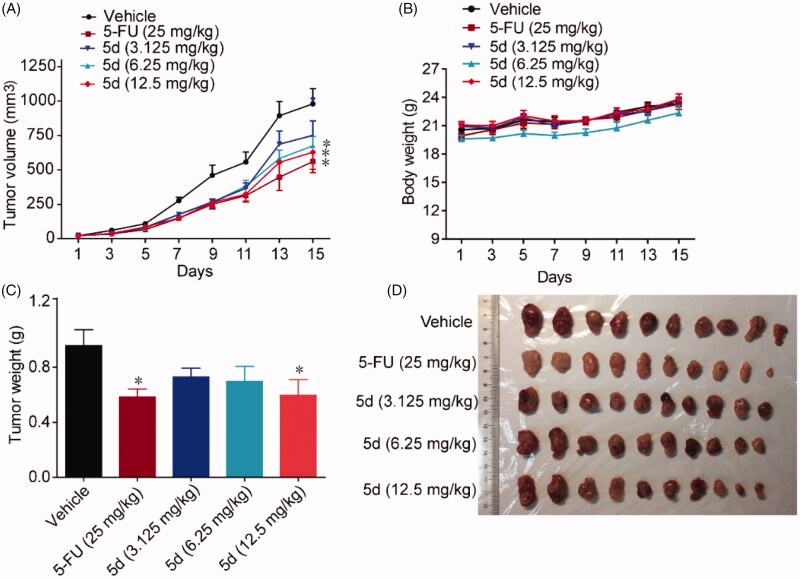
Compound **5d** suppressed colon cancer growth in immunocompetent mice. 1 × 10^6^ CT26 cells were transplanted subcutaneously into the armpit of the BALB/c mice. Three days after transplantation, mice were randomly allocated to vehicle control or treatment groups (*n* = 10). Drugs were administered on days 1–14. Tumour volume (A) and mice body weight (B) were recorded. After sacrifice, solid tumours were separated and weighed (C, D). Data represent mean ± SEM, *n* = 10, *p < 0.05, **p < 0.01 *vs* vehicle.

Given that it was the most prominent inhibitor *in vitro*, we chose compound **5d** for further pharmacodynamic evaluation *in vivo*. Bowel cancer is the third leading cause of cancer in the world, thus we first evaluated the efficacy of compound **5d** on the growth of murine CT26 colon carcinoma xenograft model in immunocompetent BALB/c mice. Fluorouracil (5-FU) is one of the most commonly used drugs to different cancers including bowel cancer, and was used as the positive control. The CT26 tumour-bearing mice were treated with **5d** (3.125, 6.25, 12.5 mg/kg, i.g, every day) or 5-FU (25 mg/kg, i.p, every other day). Tumour volumes were measured every two days for 14 days. As outlined in Figure 7, treatment of **5d** significantly suppressed the growth of tumour in a dose-dependent manner as compared with the vehicle group. Especially, **5d** reduced tumour volume by 35.9% and average tumour weight by 37.5% at the dose of 12.5 mg/kg, which were comparable to that of 5-FU. Moreover, no significant change in body weight of the mice was observed in administration groups compared with the vehicle group during the 14-day treatment.

**Figure 8.**
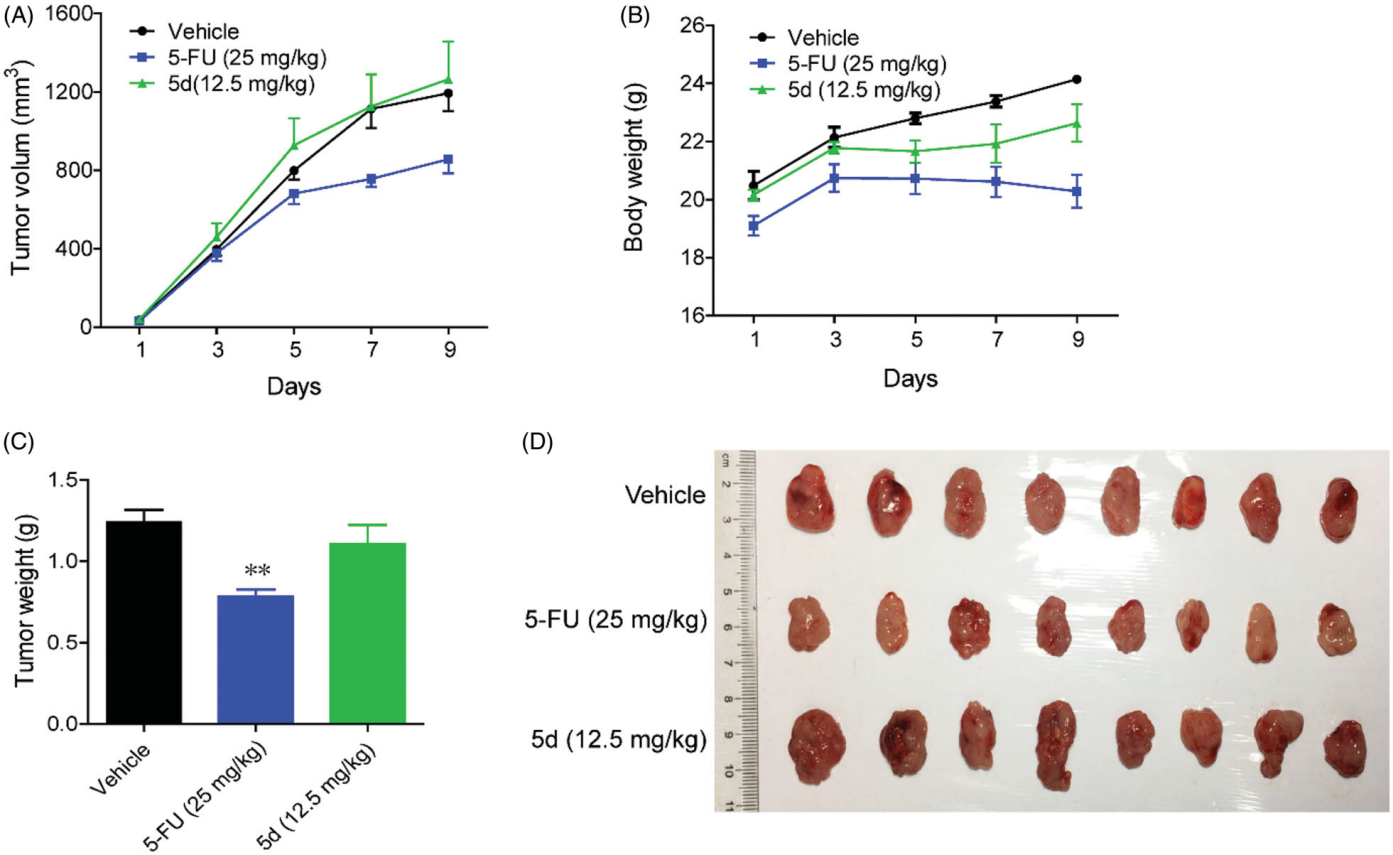
Compound **5d** did not suppress the growth of CT26 tumour in nude mice. 1 × 10^6^ CT26 cells were transplanted subcutaneously into the armpit of immunodeficient BALB/c nude mice. Three days after transplantation, mice were randomly allocated to vehicle control or treatment groups (*n* = 8). Drugs were administered on days 1–9. (A) Tumour volume and mouse body weight (B) were recorded. After sacrifice, solid tumours were separated and weighed (C, D). Data represent mean ± SEM, *n* = 8, *p < 0.05, **p < 0.01 *vs* vehicle.

Then, in order to clarify whether the *in vivo* antitumour efficacy of compound **5d** was related to the mouse immune system, we repeated the above experiment in immunodeficient nude mice. Briefly, BALB/c nude mice bearing established CT26 colon carcinoma xenograft were treated with compound **5d** and 5-FU for 10 days. As introduced in Figure 8, administration of **5d** did not block tumour growth as compared with the vehicle group, while classical cytotoxics 5-FU significantly suppressed tumour growth. Taken together, these results implied that the *in vivo* antitumour efficacy of compound **5d** was achieved by activating mouse immune system rather than its cytotoxicity.

### 3.8. Compound 5d could inhibit the growth of tumour cells by curbing proliferation and inducing apoptosis in CT26 tumour tissues

**Figure 9.**
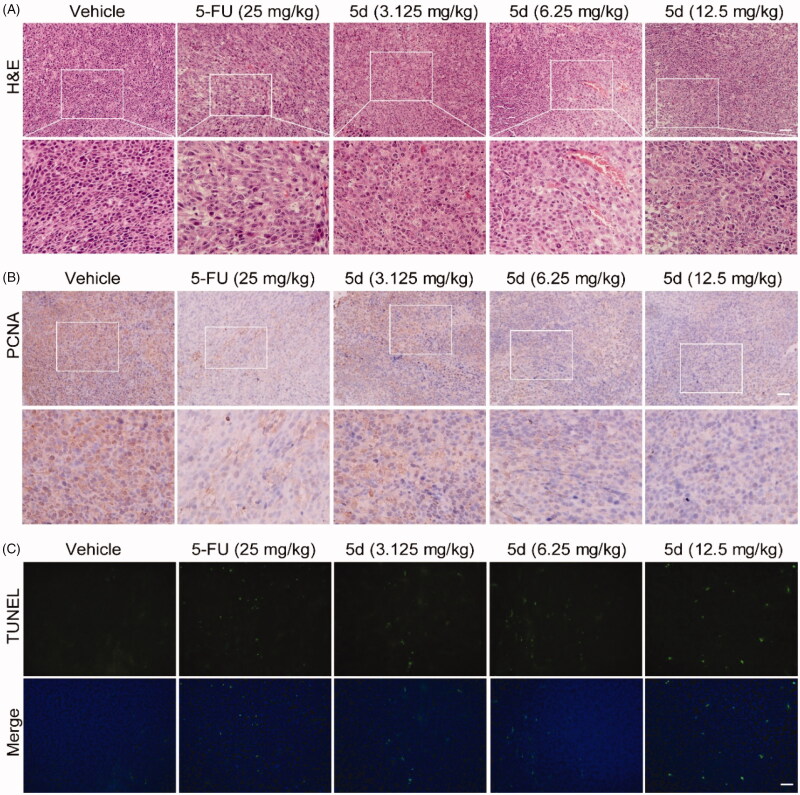
Compound **5d** dose-dependently reduced tumour cells and induced apoptosis in mice. The tumour tissues were infused in formaldehyde solution for paraffin section. (A) H&E staining of tumour tissue. (B) Expression of PCNA. (C) TUNEL staining of tumour sections. Scale bar: 50 μm.

In order to further observe morphological changes in tumour tissues after administration of compound **5d**, the tumour tissue sections obtained from the BALB/c mice bearing CT26 colorectal tumours were stained with haematoxylin-eosin (H&E) to highlight different tissue structures, such as the nucleus and cytoplasm. As illustrated in Figure 9(A), the number of tumour cells in 5-FU-treated group was significantly reduced as compared with the vehicle group. Similarly, administration of **5d** decreased the number of tumour cells, as well as led to the morphological changes including cell shrinkage and chromatin condensation in a dose-dependent manner. In addition, it was clearly observed from the enlarged local image that there are more blood vessels in the compound **5d** group, and their morphology appears more normal, suggesting that compound **5d** might contribute to tumour vascular normalisation, thereby promoting antitumour effect of the immune system^47^.

Then, to understand the mechanism of tumour cell death, we detected the level of proliferating cell nuclear antigen (PCNA) protein and tumour cell apoptosis by immunohistochemistry (IHC) and terminal deoxynucleotidyl transferase dUTP nick end labelling (TUNEL) experiments, respectively. PCNA is known as a molecular marker for assessing cell proliferation status, and TUNEL assay is the most widely used method to identify apoptotic cells. The results of IHC analysis showed that the expression of PCNA protein in the 5-FU group was significantly reduced as compared with the vehicle group. The similar results were observed in the **5d** treatment group and showed a dose-response relationship (Figure 9(B)). In addition, the results of TUNEL assay showed that the tumour cells had less apoptosis in the vehicle group, while the apoptosis increased in the 5-FU group. Likewise, treatment with **5d** also significantly induced the tumour cell apoptosis in a dose-dependent manner (Figure 9(C)). These data revealed that compound **5d** displayed *in vivo* antitumour efficacy by inhibiting tumour cell proliferation and inducing apoptosis.

**Figure 10.**
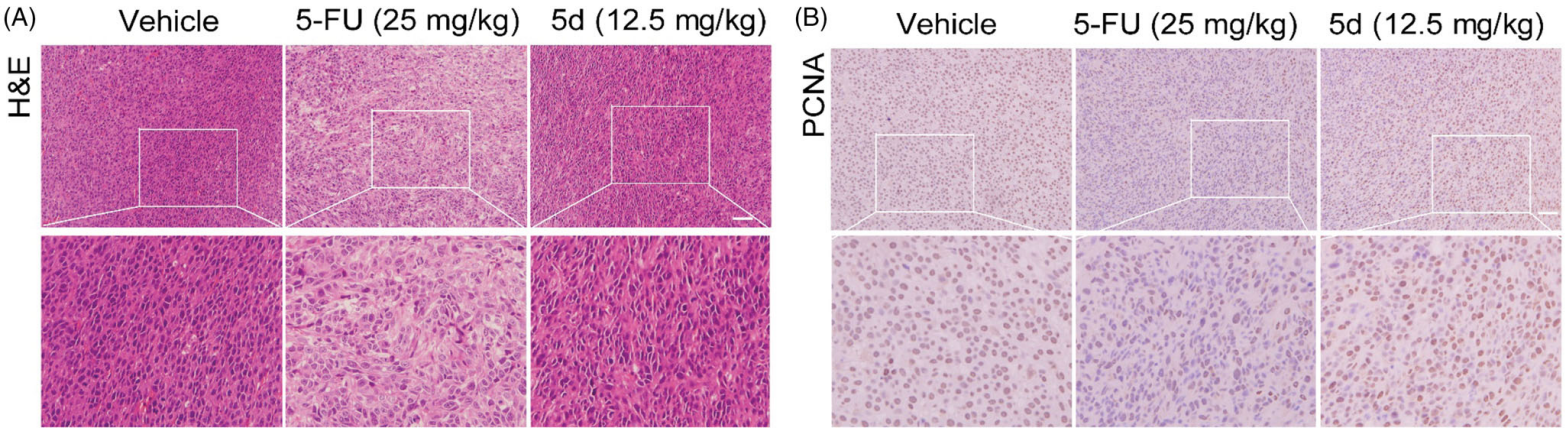
Compound **5d** could not suppress the proliferation of tumour cells in immunodeficient mice. Tumour sections were infused in formaldehyde solution for immunohistochemistry. (A) H&E staining of tumour tissue. (B) Expression of PCNA. Scale bar: 50 μm.

To verify the above conclusions, we further performed H&E and IHC staining on the nude mouse CT26 tumour tissues. As depicted in Figure 10(A), the tumour cells in the **5d**-treated group remained intact morphology and were arranged in an orderly manner as that in the vehicle-treated group, while the results of 5-FU treatment were just the opposite. Moreover, the results of TUNEL assay showed that administration of **5d** had no influence on the expression of PCNA (Figure 10(B)). These findings further supported the above conclusions.

### 3.9. Compound 5d facilitated immune system rejuvenation in CT26 tumour-bearing mice

**Figure 11.**
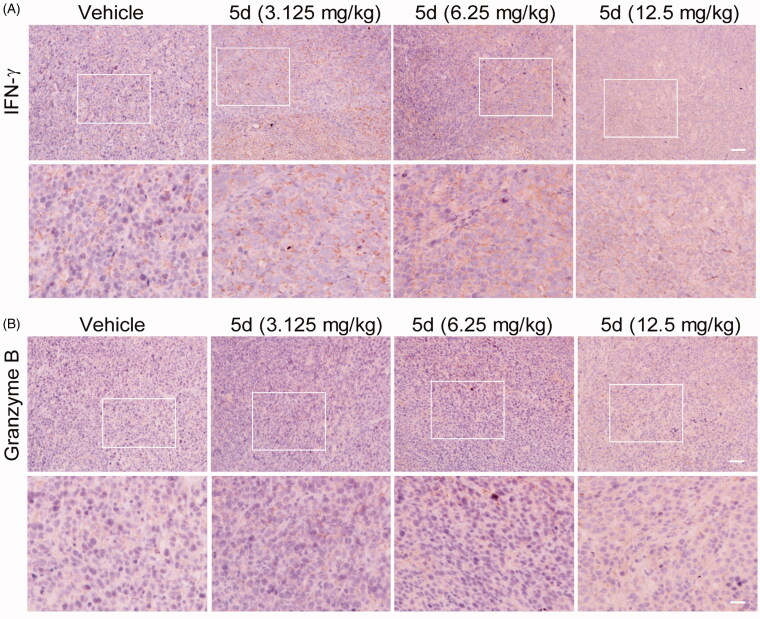
Compound **5d** up-regulated the expression of IFN-*γ* and granzyme B. Tumour sections were infused in formaldehyde solution for immune stain. (A) Expression of IFN-*γ*. (B) Expression of Granzyme B. Scale bar: 50 μm.

To investigate the effect of compound **5d** on the immune function in CT26 tumour-bearing mice, we carried out the IHC experiment to detect the expression of IFN-*γ* and granzyme B in the tumour tissues. IFN-*γ* is a key cytokine secreted primarily by activated T cells and NK cells, which is essential for both innate and adaptive immunity. Therefore, IFN-*γ* is an important sign of rejuvenation of the immune system. Moreover, cytotoxic T lymphocytes (CTLs) is the key effector cells of the adaptive antitumour immune response. Granzyme B is the primary mediator of apoptosis by CTLs and NK cells. It can initiate the processing of caspases and apoptosis when it is released from activated CTLs^48^. As detailed in Figure 11, compared with the vehicle, administration of compound **5d** increased the expression of IFN-*γ* in a dose-dependent manner in the CT26 tumour tissues, suggesting that **5d** treatment could facilitate T cell activation and the rejuvenation of the immune system in the tumour microenvironment. Similarly, all treatments of **5d** elevated granzyme B levels, which was consistent with the results of the TUNEL assay described above.

**Figure 12.**
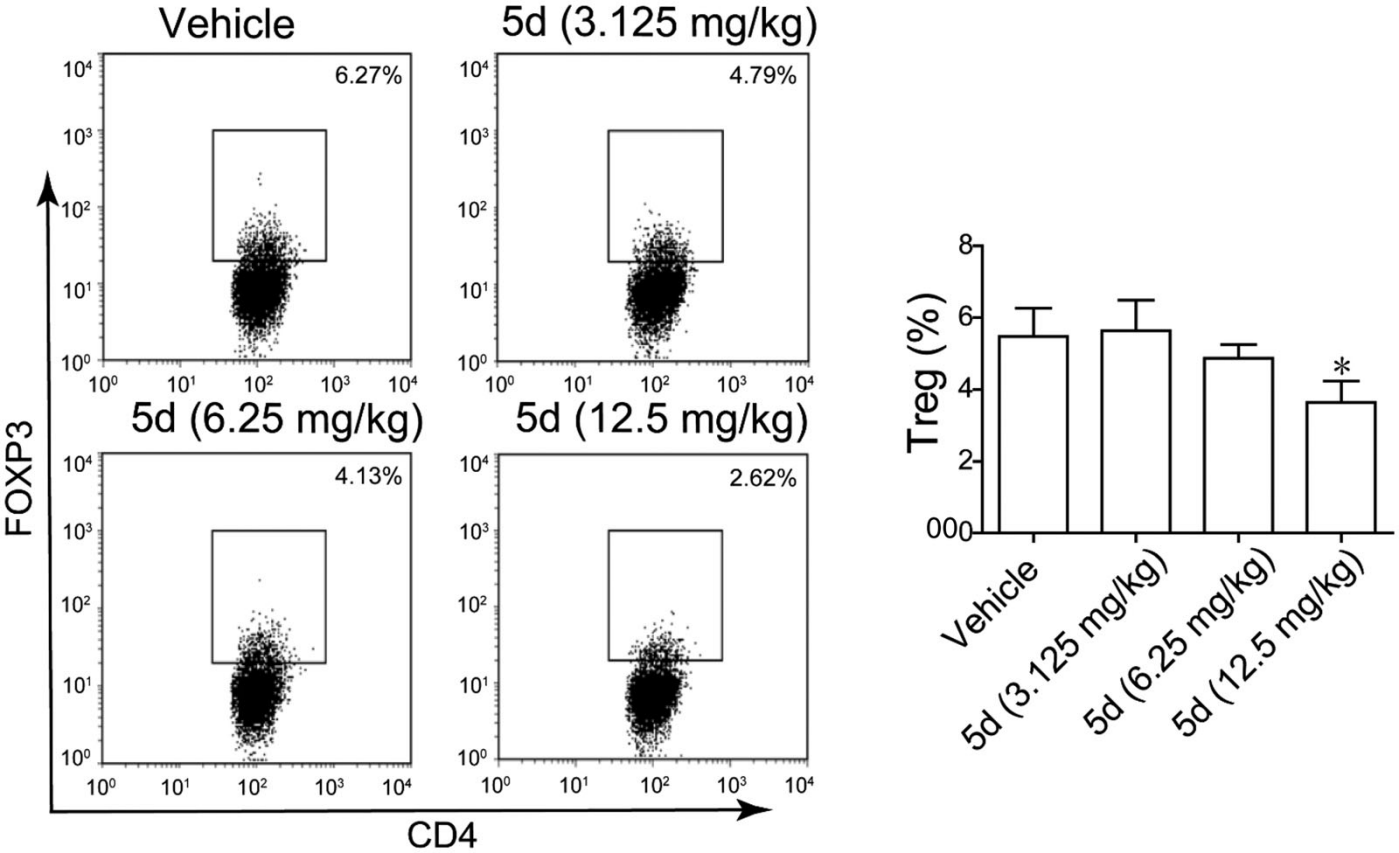
Compound **5d** decreased Treg cell differentiation in the CT26 tumour-bearing mouse. (A) Decreased differentiation of Treg cells in the spleen of tumour-bearing mice treated by compounds. (B) Statistical analysis of result A. Data represent mean ± SEM, *p < 0.05 *vs* vehicle.

Furthermore, we also detected the content of FoxP3^+^ Treg cells in the CT26 tumour-bearing mouse spleens by fluorescence activated cell sorting (FACS) assay (Figure 12). The results showed that **5d** treatment decreased the number of FoxP3^+^ Treg cells in a dose-dependent manner. Especially, the amount in the high dose group was reduced 2.4-fold compared with the vehicle group. Taken together, these results revealed that the compound **5d** could promote T cell activation, decrease FoxP3^+^ Treg cell population and heighten the function of CTLs.

### 3.10. Compound 5d could effectively suppress melanoma growth in immunocompetent mice

**Figure 13.**
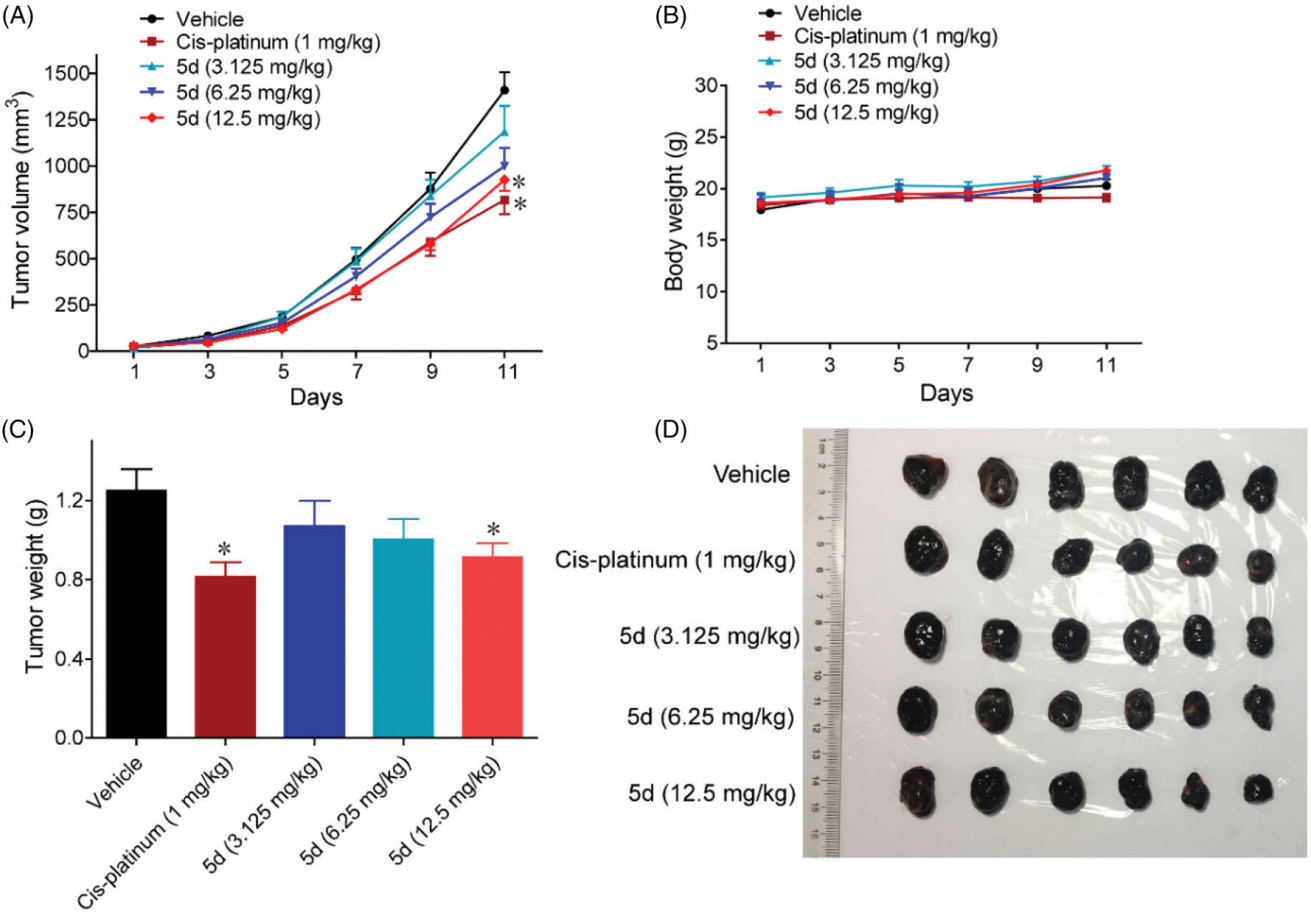
Compound **5d** suppressed melanoma growth in mice. 1 × 10^6^ melanoma cells were transplanted subcutaneously into the armpit of the BALB/c mice. Three days after transplantation, mice were randomly allocated to vehicle control or treatment groups (*n* = 6). Drugs were administered on days 1–11. (A) Tumour volume and mouse body weight (B) were recorded. After sacrifice, solid tumours were separated and weighed (C, D). Data represent mean ± SEM, *n* = 6, *p < 0.05, **p < 0.01 *vs* vehicle.

In addition, we also utilised a B16F1 melanoma mouse xenograft model to observe the efficacy of compound **5d** on the other tumours. Malignant melanoma is a highly invasive tumour and its immunotherapy has been receiving attention in recent years. As mentioned above, B16F1 is a murine melanoma cell line that expresses IDO1. The mouse xenograft model was established by subcutaneously implanting B16F1 melanoma cells into BALB/c mice and treated with **5d** (3.125, 6.25, 12.5 mg/kg, i.g, every day) or the positive control Cis-platinum (1 mg/kg, i.p, every other day). Tumour volumes were measured every two days for 11 days. As shown in Figure 13, treatment with **5d** significantly suppressed the growth of tumour in a dose-dependent manner both in tumour volume and in tumour weight as compared with the vehicle group without significant body weight loss. Especially at high dose, the average volume of tumour was reduced by 34.3% after **5d** treatment, which were comparable to Cis-platinum.

**Figure 14.**
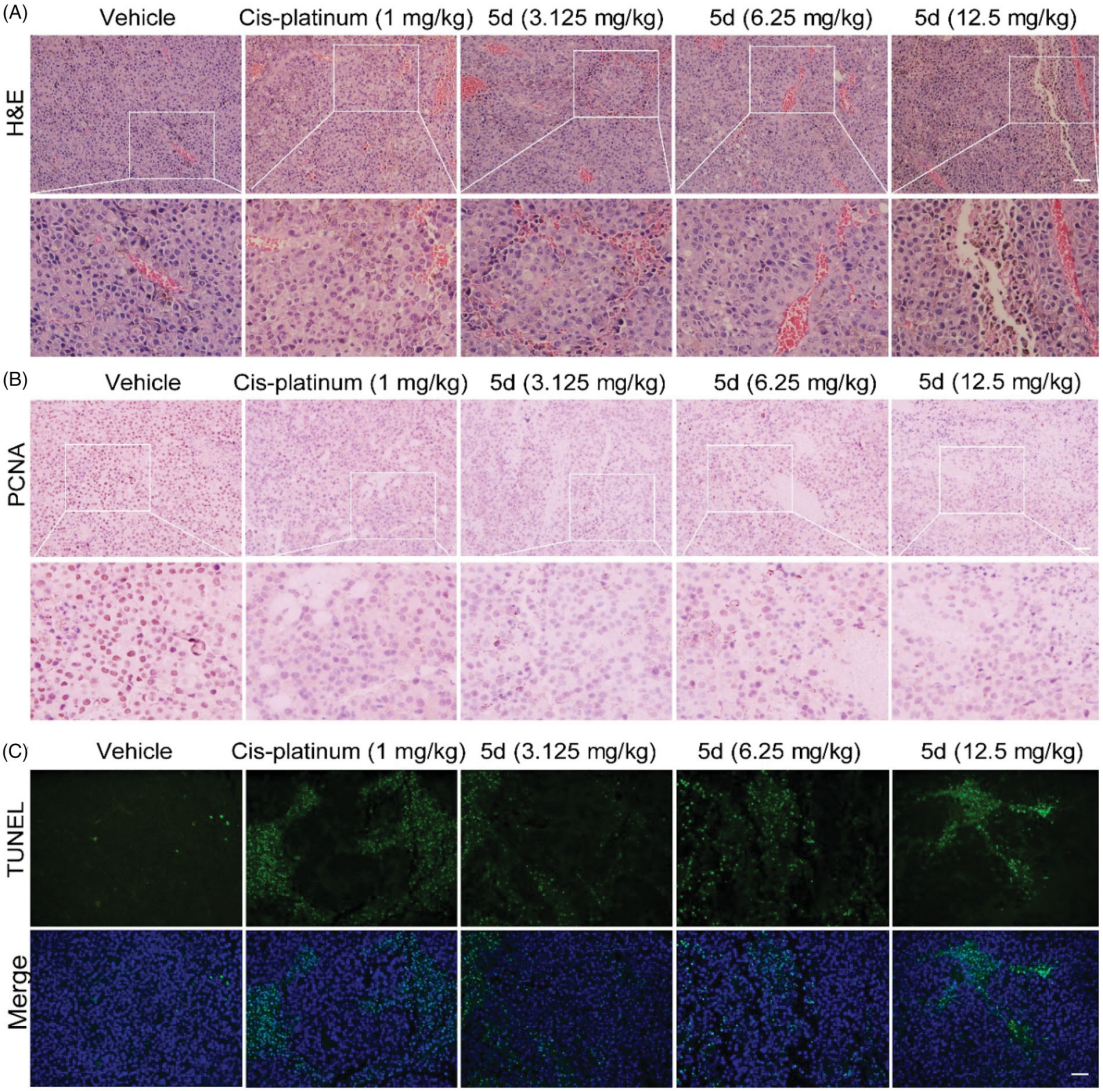
Compound **5d** dose-dependently reduced tumour cells and induced apoptosis in mice. Tumour sections were infused in formaldehyde solution for immunohistochemistry. (A) H&E staining of tumour tissue. (B) Expression of PCNA. (C) TUNEL staining of tumour sections. Scale bar: 50 μm.

**Figure 15.**
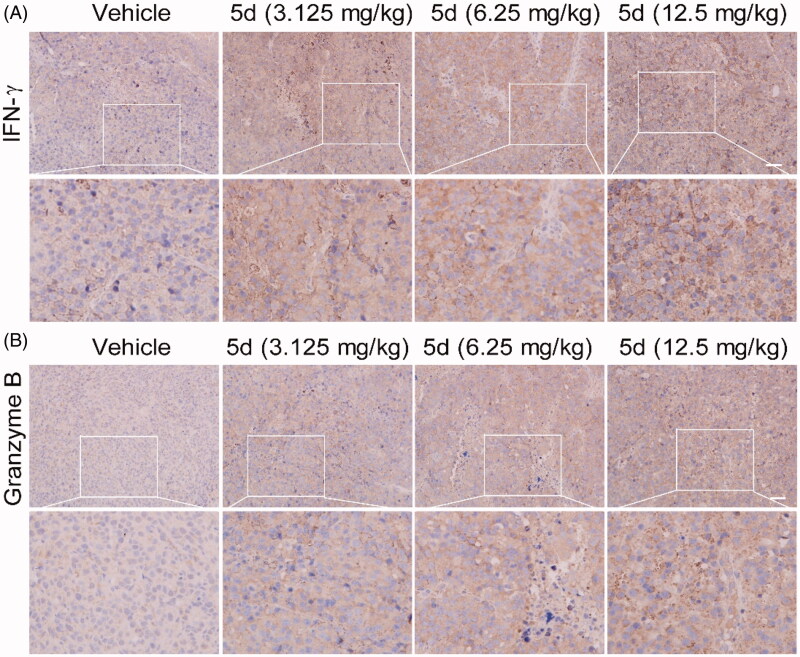
Compound **5d** promoted T cells activation while reduced Foxp3^+^ Treg cell number in tumour tissue. Tumour sections were infused in formaldehyde solution for immune stain. (A) Expression of IFN-*γ*. (B) Expression of Granzyme B. Scale bar: 50 μm.

Analogously, we also performed H&E, IHC and TUNEL experiments on the B16F1 melanoma tissues (Figure 14 and 15). These results indicated that compound **5d** could effectively contribute to the morphological changes of B16F1melanoma cells, reduce the number of tumour cells by inhibiting proliferation and inducing apoptosis, and facilitate tumour vascular normalisation. Importantly, compound **5d** also promoted T cell activation by up-regulating IFN-*γ* expression, and elevated CTLs function by stimulating granzyme B secretion. These results reaffirm that compound **5d** could inhibit tumour growth by activating the mouse immune system.

### 3.11. Molecular modelling studies

**Figure 16.**
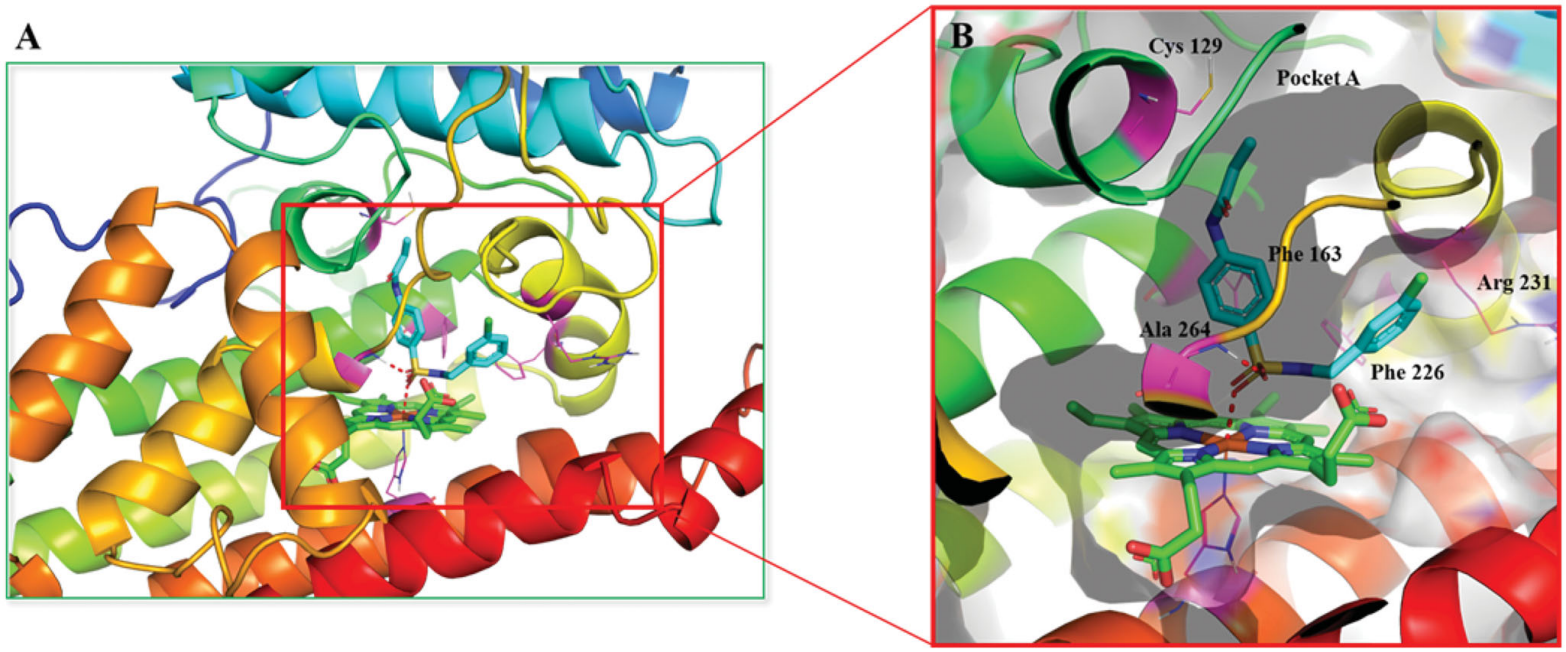
(A) Proposed binding mode of compound **5d** docked into the crystal structure of human IDO1 (PDB: 4PK5) by IFD methods. (B) The interactions between compound **5d** and IDO1. Polar interactions were presented by red dotted line.

Based on their outstanding performance in biological activity, we performed computational studies to elucidate the interactions of compounds **5d** with IDO1. Since the high flexibility of the side chain and backbone of IDO1 protein, the classical semi-flexible docking method often poses a problem for IDO1 docking, whose algorithm relies on a rigid protein structure. Thus, the induced fit docking (IFD) method was utilised to overcome the difficulty mentioned above. The cocrystallization of Amg-1 in complex with human IDO1 protein (PDB ID: 4PK5) was employed as the template in the following docking calculations because of its relatively high resolution and more structural information at pocket B. As shown in Figure 16, The benzsulfamide moiety situated deeply in pocket A and their benzene ring was interacted with Phe163 through π-π interaction, while the benzylamine moiety extended towards pocket B formed by Phe163, Phe226, Arg231, Leu234 and Ile354. Specifically, one oxygen atom of the sulphonyl group bond directly to the haem iron of IDO1 while the other oxygen atom formed a hydrogen bond with the main chain NH group of Ala264. Acryloyl groups located at the top of pocket A, and ethenyl moiety of acryloyl group extended towards a small hydrophobic subpocket formed by Met88, Leu124, Cys129, Leu234 and Gly262. This computational study finds that the occupation of the small hydrophobic subpocket in pocket A may a key factor affecting IDO1 inhibitory activity. These data could be useful for further optimisation.

## 4. Conclusions

In this study, the hit compound BS-1 was found as an IDO1 inhibitor in-house compound library. After structural optimisation, a series of sulphonamide derivatives were designed, synthesised, and evaluated in the HeLa cell-based IDO1/kynurenine assay, resulting in the identification of secondary sulphonamides as novel IDO1 inhibitors. Among them, the compounds **5d**, **5l** and **8g** displayed the strongest inhibitory effect, and their activities were significantly improved over the hit compound BS-1. The *in vitro* results showed that these compounds could bind to IDO1 protein in HeLa cells without affecting the cell viability and the IDO1 protein expression under experimental conditions, indicating that these compounds blocked the kynurenine pathway by influencing IDO1 enzyme activity rather than IDO1 expression and/or cell viability. In addition, these compounds could promote the T cell proliferation and suppress the differentiation of naïve CD4^+^ T cell into FoxP3^+^ Treg cells, suggesting that these sulphonamides could reverse IDO1-mediated immunosuppression.

Based on its excellent performance *in vitro*, compound **5d** was selected for further evaluating the pharmacodynamic effects in three types of tumour-bearing mouse models, including two immunocompetent mouse xenografts and one immunodeficient mouse xenograft. These *in vivo* experiments demonstrated that treatment with **5d** could effectively inhibit the growth of murine CT26 coloreatic carcinoma xenograft tumour in a dose-dependent manner in immunocompetent mice, but not in immunodeficient mice, which implied that the *in vivo* efficacy of compound **5d** was closely associated with the mouse immune system. Similarly, **5d** also significantly blocked the growth of B16F1 melanoma in immunocompetent mice. Functionally, biochemical experiment and pathological analysis were utilised to detect these tumour tissues. The results from two immunocompetent mouse models indicated that compound **5d** could induce the morphological changes of both CT26 and B16F1 tumour cells, decrease the number of tumour cells by suppressing proliferation and inducing apoptosis. Importantly, compound **5d** could contribute to T cell activation by stimulating IFN-*γ* expression, improve CTLs function by inducing granzyme B secretion, and inhibit FoxP3^+^ Treg cell differentiation, which would help facilitate the rejuvenation of the immune system. These promising results motivate us to further develop this new type of IDO1 inhibitors.
